# Expansion of commensal fungus *Wallemia mellicola* in the gastrointestinal mycobiota enhances the severity of allergic airway disease in mice

**DOI:** 10.1371/journal.ppat.1007260

**Published:** 2018-09-20

**Authors:** Joseph H. Skalski, Jose J. Limon, Purnima Sharma, Matthew D. Gargus, Christopher Nguyen, Jie Tang, Ana Lucia Coelho, Cory M. Hogaboam, Timothy R. Crother, David M. Underhill

**Affiliations:** 1 F. Widjaja Foundation Inflammatory Bowel & Immunobiology Research Institute, and the Division of Immunology, Department of Biomedical Sciences, Cedars-Sinai Medical Center, Los Angeles, California, United States of America; 2 Division of Pulmonary and Critical Care Medicine, Department of Medicine, Mayo Clinic, Rochester, Minnesota, United States of America; 3 Genomics Core, Department of Biomedical Sciences, Cedars-Sinai Medical Center, Los Angeles, California, United States of America; 4 Women’s Guild Lung Institute, Department of Medicine, Cedars-Sinai Medical Center, Los Angeles, California, United States of America; 5 Division of Pediatric Infectious Diseases, Department of Medicine, and the Division of Immunology, Department of Biomedical Sciences, Cedars-Sinai Medical Center, Los Angeles, California, United States of America; Memorial Sloan-Kettering Cancer Center, UNITED STATES

## Abstract

The gastrointestinal microbiota influences immune function throughout the body. The gut-lung axis refers to the concept that alterations of gut commensal microorganisms can have a distant effect on immune function in the lung. Overgrowth of intestinal *Candida albicans* has been previously observed to exacerbate allergic airways disease in mice, but whether subtler changes in intestinal fungal microbiota can affect allergic airways disease is less clear. In this study we have investigated the effects of the population expansion of commensal fungus *Wallemia mellicola* without overgrowth of the total fungal community. *Wallemia* spp. are commonly found as a minor component of the commensal gastrointestinal mycobiota in both humans and mice. Mice with an unaltered gut microbiota community resist population expansion when gavaged with *W*. *mellicola*; however, transient antibiotic depletion of gut microbiota creates a window of opportunity for expansion of *W*. *mellicola* following delivery of live spores to the gastrointestinal tract. This phenomenon is not universal as other commensal fungi (*Aspergillus amstelodami*, *Epicoccum nigrum*) do not expand when delivered to mice with antibiotic-depleted microbiota. Mice with *Wallemia*-expanded gut mycobiota experienced altered pulmonary immune responses to inhaled aeroallergens. Specifically, after induction of allergic airways disease with intratracheal house dust mite (HDM) antigen, mice demonstrated enhanced eosinophilic airway infiltration, airway hyperresponsiveness (AHR) to methacholine challenge, goblet cell hyperplasia, elevated bronchoalveolar lavage IL-5, and enhanced serum HDM IgG1. This phenomenon occurred with no detectable *Wallemia* in the lung. Targeted amplicon sequencing analysis of the gastrointestinal mycobiota revealed that expansion of *W*. *mellicola* in the gut was associated with additional alterations of bacterial and fungal commensal communities. We therefore colonized fungus-free Altered Schaedler Flora (ASF) mice with *W*. *mellicola*. ASF mice colonized with *W*. *mellicola* experienced enhanced severity of allergic airways disease compared to fungus-free control ASF mice without changes in bacterial community composition.

## Author summary

The microbiome is the ecosystem of bacteria, fungi, viruses, and other microorganisms that live in and on us. The intestines are where most of these organisms reside, and the composition of the intestinal microbiota has a profound effect on immune system function throughout the body. In this manuscript, we observe that expansion of a certain species of house dust fungus (*Wallemia mellicola*) can occur in the intestines of mice after they are treated with antibiotics and exposed to the fungus, but that mice with an intact and healthy intestinal microbiota resist this expansion. After expansion of this fungal population, the mice are more prone to develop asthma-like inflammation in their lungs when exposed to allergens. Although it is not known whether the same phenomenon can occur in people with asthma, we have also identified this fungus as a component of the microbiota of some healthy humans. Elimination of this organism from the intestines is not as simple as taking an antifungal medication because antifungal medications can also disrupt the balance of the intestinal microbial community and may similarly worsen allergic airways disease.

## Introduction

The gut microbiome is a dynamic ecosystem that profoundly influences immune function throughout the body [1–3]. Commensal microorganisms are recognized by the host immune system and can alter systemic immune response or produce bioactive metabolites which are absorbed into the bloodstream and have pharmacological effect on distant organ systems [4–7]. The commensal microbial composition of the gut can therefore have a distant effect on immune function in the lung and other organ systems; this is the concept of the gut-lung axis.

In addition to bacteria, the healthy gastrointestinal system contains a community of commensal fungi that live in the gut and continually interact with the gastrointestinal mucosa [8–10]. As many commensal fungi require fastidious culture conditions, non-culture-based assays are essential to comprehensively profile intestinal fungal communities. Like 16S sequencing of bacterial DNA, targeted amplicon sequencing of the first internally transcribed spacer region (ITS1) of the fungal ribosomal RNA genome can be used to generate a profile of the fungal organisms in a sample and their relative abundance [11]. Although there are several sample processing and analysis considerations distinct from 16S analysis, ITS1 sequencing has been used successfully to profile gastrointestinal fungal communities in a both mice and humans [9, 11]. ITS1 sequences have high variability across the phylogenetic tree and can generally be used to classify organisms to the genus and often species level. Studies using ITS1 sequencing to profile the human gastrointestinal mycobiota generally report a diverse community containing more than 50 unique genera with substantial variation across individuals [8].

Fungi are particularly important in asthma. Inhaled fungi are a well-described trigger for asthma, and patients with fungal sensitization have increased asthma incidence and more severe and fatal disease compared to patients sensitized to other allergens [12, 13]. Interestingly, many commensal fungal species commonly found in the human gastrointestinal tract are triggers of allergic respiratory illness when inhaled such as *Aspergillus*, *Cladosporium*, *Penicillium*, and others [14]. We know that avoidance of airborne fungi can improve control of asthma in some patients, but it is uncertain whether variation in the commensal fungal species which reside in the gut and continually interact with the host immune system may also alter the severity of allergic airways disease. This is an emerging area of investigation, but tantalizingly, one recent study of human infants suggested that gut fungal dysbiosis may be more strongly associated with the development of allergic wheeze than bacterial dysbiosis [15].

Prior studies of the intestinal mycobiota have largely focused on *Candida albicans* and other *Candida* species. These are opportunistic pathogens that are recognized to be subject to intestinal overgrowth after exposure to oral antibacterials. In mouse models, intestinal overgrowth of *Candida* can be induced after treatment with the antibiotic cefoperazone and exposure to a bolus of live yeasts [16]. Mice with *Candida* overgrowth have exacerbated allergic airways disease, which has been suggested to occur via fungal secretion of prostaglandins that are absorbed into systemic circulation [4]. Building on these studies, we previously investigated whether suppressing natural commensal fungal populations with anti-fungal drugs could suppress allergic airway disease. We found, instead, that antifungal treatment surprisingly exacerbated allergic airways disease [17]. Although anti-fungal therapy depleted native *Candida* populations, ITS1 sequencing analysis of their gastrointestinal mycobiota suggested that other, relatively drug-resistant fungal organisms increased in abundance; specifically, *Aspergillus amsteoldami*, *Wallemia mellicola*, and *Epicoccum nigrum*. Further, when administered together as a cocktail by oral gavage, these three fungi exacerbated allergic airways disease. These studies have led us now to investigate the idea that relatively modest changes in naturally occurring non-*Candida* intestinal fungi may influence pulmonary immune responses.

In this manuscript, our experimental results have led us to focus on *Wallemia mellicola*, a common environmental fungus. *W*. *mellicola* is a ubiquitous spore forming filamentous basidiomycete that is a common component of house dust and an agent of food spoilage [18–21] (S1A and S1B Fig). *W*. *mellicola* is slow growing and may therefore be less commonly detected by culture than other commensal fungi such as *Candida* and *Aspergillus*. *Wallemia* spp. are highly xerotolerant and have been reported to secrete several toxins when grown in culture such as walleminol, walleminone, and wallimidione [22–24]. The metabolic products generated by *Wallemia* residing in the mammalian intestine are unknown. *W*. *mellicola* does not generally act as a human pathogen, but there are some reported associations between *Wallemia* exposure and lung disease. Specifically, asthmatic patients have a high incidence of immune sensitization to *Wallemia*, and *Wallemia* was identified as one of a handful of environmental fungi associated with increased risk of asthma in individuals living in water damaged homes [25, 26].

## Results

### Enhanced colonization with *Wallemia mellicola*

**Fig 1:**
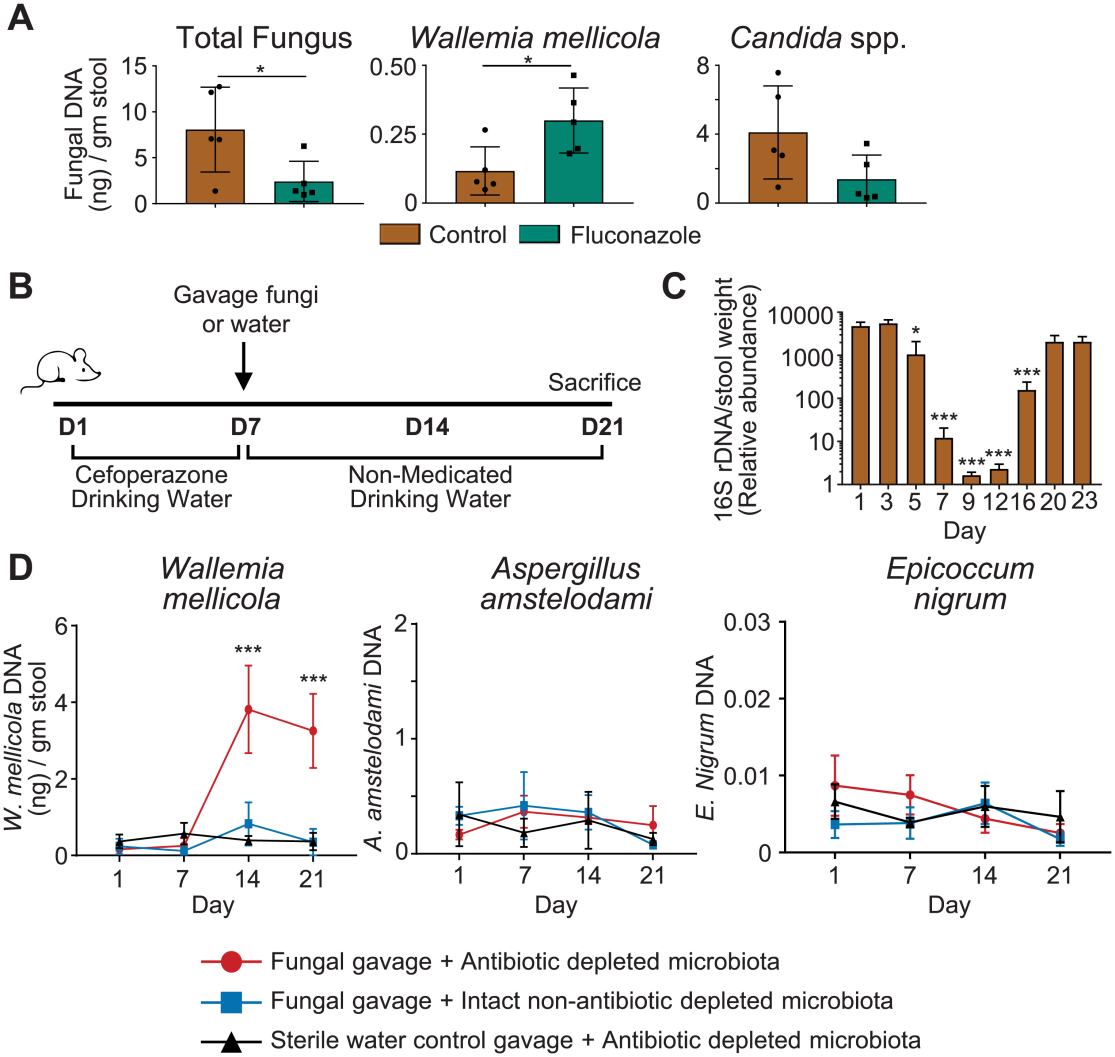
Enhanced colonization with *Wallemia mellicola*. (**A**) Fluconazole in the drinking water alters but does not uniformly deplete gastrointestinal commensal fungal populations. Levels of the indicated organisms in fecal samples from specific pathogen free (SPF) mice treated for 21 days with fluconazole were measured by quantitative PCR. (**B**) Graphical depiction of fungal colonization protocol utilized in experiments in panel D. (**C**) Antibiotic (cefoperazone) treatment in the drinking water profoundly and transiently depletes total stool bacterial content (measured by quantitative 16S PCR) in control animals over the course of an experiment. Antibiotic water in this experiment administered day 3–12. Statistical comparisons are between indicated bar and day #1 samples. (**D**) Mice with antibiotic-depleted gastrointestinal flora are vulnerable to *Wallemia mellicola* expansion. Mice were exposed by gastric gavage to the indicated fungi (5 x 10^6^ live organisms), and stool levels were assessed by quantitative PCR at the indicated times. Figures are a representative example of experiment that was independently performed three times with 5 animals per group. *, p < .05; **, p < .01; ***, p<0.001; unpaired two-tailed Student’s T-test for comparison of two groups (1A, 1C); One-way ANOVA for comparison of three groups (1D).

We have previously observed that a fluconazole-induced intestinal fungal dysbiosis state enhances the severity of allergic airways disease in mice [17]. Fluconazole therapy has complex and surprising effects on commensal gut fungal communities. Although fluconazole moderately depletes the overall burden of commensal fungi including *Candida* spp., fungi that are relatively resistant to fluconazole expand in population (Fig 1A). Notably, this is not simply an increased relative abundance due to mismatched decline in individual species abundance compared to total fungal burden. Rather, the absolute quantity of selected gut fungal species can increase during fluconazole therapy. Our prior observation that fluconazole depletion of gut mycobiota exacerbates the severity of allergic airways disease suggests that previously-characterized effects of intestinal *Candida* overgrowth on allergic airways disease may not be restricted to *Candida* [17]. To investigate further, we first sought to define conditions that generate a fungal dysbiosis in the gut by means other than inducing *Candida* overgrowth or repeated oral gavage with fungi.

We examined conditions required for enhancing intestinal colonization with several non-*Candida* commensal fungal species that are natively found in the intestinal microbiota of specific pathogen free mice (SPF) at our animal facility: *Wallemia mellicola*, *Aspergillus amstelodami*, *and Epicoccum nigrum*. These were selected because we have previously observed that these species expand in relative population abundance in our fluconazole treated mice who develop exacerbated sensitivity to the house dust mite model of asthma [17]. We initially attempted simply a gavage of high dose of cultured live fungi from each species into the mouse gastrointestinal tract. This did not result in sustained expansion of the population of any fungus. We hypothesized that resistance to expansion of the fungal populations was due to competition from other commensal gastrointestinal microbes. Previous authors have shown that mice with an intact commensal bacterial community resist colonization with *Candida albicans*, but that antibiotic depletion of mouse commensal bacteria renders them vulnerable to *C*. *albicans* overgrowth after exposure [27]. We therefore performed 7 days of treatment of mice with cefoperazone followed by one-time gavage of each fungus (Fig 1B). Cefoperazone treatment had a devastating effect on bacterial communities, depleting total commensal bacterial burden of the gut by nearly 10,000x fold, but gut bacterial abundance recovered to previous levels within 8 days of cessation of antibiotics (Fig 1C).

A single gavage of *W*. *mellicola* into a mouse with antibiotic depleted bacterial microbiota resulted in sustained and substantial increase in the population of *Wallemia* above baseline (Fig 1D). The *Wallemia*-expanded mycobiota persisted after discontinuation of antibacterials and without any further *W*. *mellicola* gavage to support the population. *W*. *mellicola* is present as a minor component of the commensal mycobiota in our mice at baseline, but mice treated with cefoperazone and gavaged with sterile water (Fig 1D) did not experience expansion of this fungus suggesting that both antibacterial depletion and exposure to a bolus of *W*. *mellicola* are necessary to generate a *Wallemia*-expanded dysbiosis state. To be certain that *W*. *mellicola* can survive and grow in the mouse intestines, we further colonized germ-free mice with *W*. *mellicola* and observed that we could culture organisms from the stool after 10 days (S1C Fig). Antibiotic treatment was not similarly sufficient to allow for enhanced colonization with *Epicoccum nigrum* or *Aspergillus amstelodami*, suggesting that antibiotic depletion of gut bacteria is not universally able to facilitate expansion of a commensal fungal species (Fig 1D). These organisms may not directly compete with bacteria for their gut ecological niche, or they may compete with bacteria that are not affected by cefoperazone therapy.

### Characteristics of the *Wallemia*-enhanced colonization state

**Fig 2:**
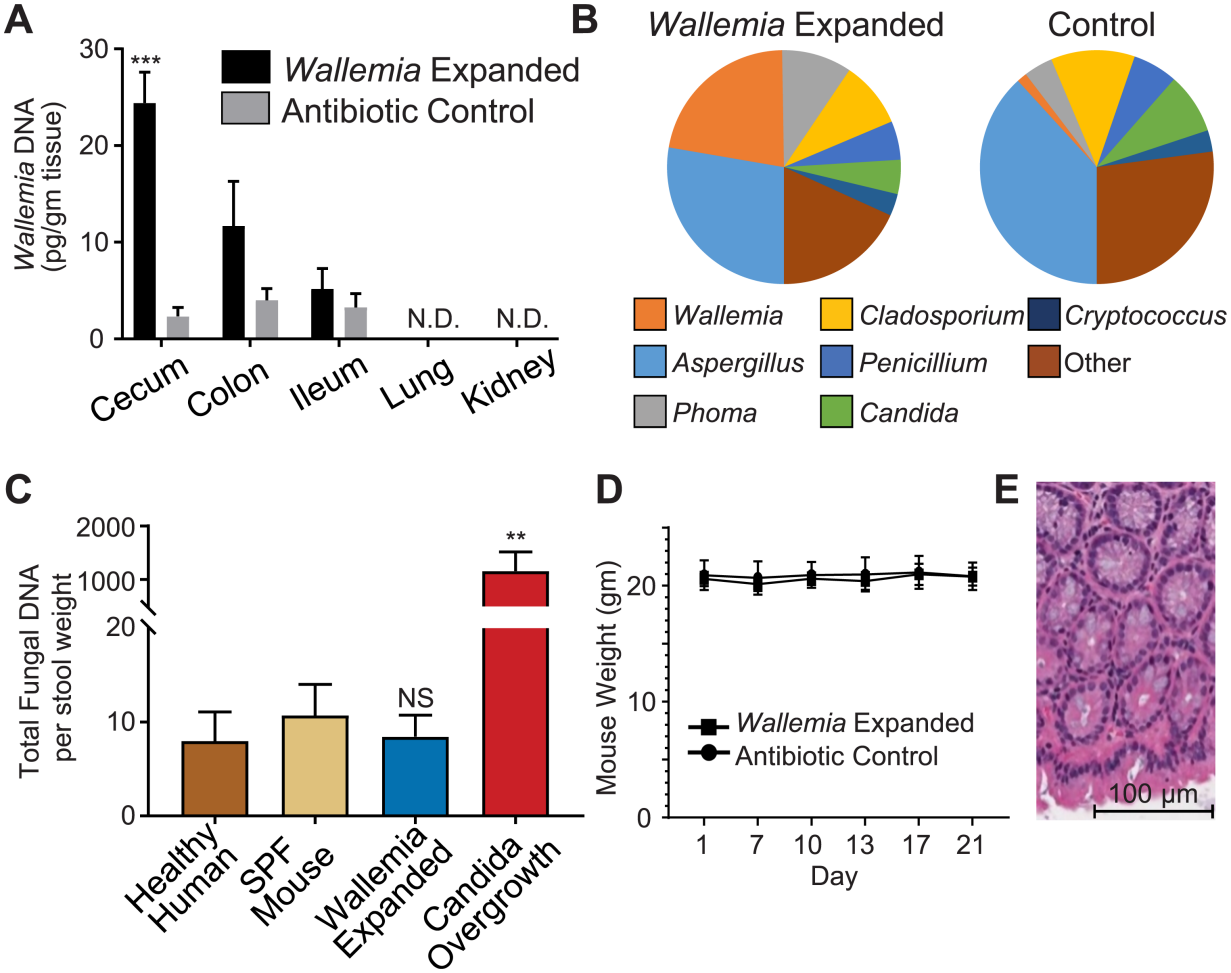
Characteristics of *Wallemia mellicola* expanded colonization. (**A**) *W*. *mellicola* population expansion occurs predominantly in the cecum and colon with no detectable *Wallemia* DNA in extraintestinal organs. The indicated tissue samples collected from antibiotic-only control or *W*. *mellicola*-colonized mice (at day 14, n = 5 per group) were analyzed for *Wallemia* DNA by quantitative PCR. (**B**) Fungal communities were assessed by ITS1 sequencing. Fecal samples from antibiotic-only control or *W*. *mellicola*-colonized mice (at day 14, n = 4 per group) were analyzed, and pie charts illustrate genera distributions. (**C**) Unlike antibiotic-associated *Candida albicans* overgrowth, total fungal burden in *Wallemia*-expanded mice does not increase compared to SPF mice. Fecal samples from the indicated sources (mice at day 14, n = 4 per group) were assessed for total fungal content by quantitative PCR. Statistical comparisons are between indicated bar and SPF mouse group. (**D-E**) *W*. *mellicola* does not induced pathology. Weight was monitored in control and *W*. *mellicola*-colonized mice (D). Histology of colons of *Wallemia*-colonized animals examined by H&E staining (400X) do not show obvious signs of inflammatory disease. Figures are representative examples of experiments independently performed at least three times. *, p < .05; **, p < .01; ***, p<0.001; unpaired two-tailed Student’s T-test.

We next explored the characteristics of the *Wallemia*-expanded mycobiota. *W*. *mellicola* colonization was predominantly in the cecum and colon and restricted to the gastrointestinal tract with no *W*. *mellicola* detected in extra-gastrointestinal organ systems (Fig 2A). Notably, we did not detect *W*. *mellicola* by rtPCR or culture in mouse lungs. Total gastrointestinal fungal abundance in mice with *Wallemia*-expanded microbiota remained similar to untreated mice, but the population of fungi had shifted such that *W*. *mellicola* had markedly increased in relative abundance (Fig 2B and 2C). This contrasts with the *Candida albicans* overgrowth state where the total fungal burden in the gastrointestinal tract increases by orders of magnitude after mice are subjected to a similar protocol of cefoperazone depletion of gut bacteria followed by *C*. *albicans* gavage (Fig 2C). Finally, there was no evidence to suggest that *W*. *mellicola* was behaving as an infectious pathogen in these mice. Specifically, mice with *Wallemia*-expanded mycobiota showed no weight loss, behavioral changes, or stool changes throughout the experiments, and there was no histological evidence of colonic inflammation (Fig 2D and 2E). Together, these data suggest that stable *W*. *mellicola* colonization is best thought of as a model of altered intestinal fungal community rather than gastrointestinal fungal overgrowth.

### *Wallemia mellicola* is found in the human gastrointestinal tract

**Fig 3:**
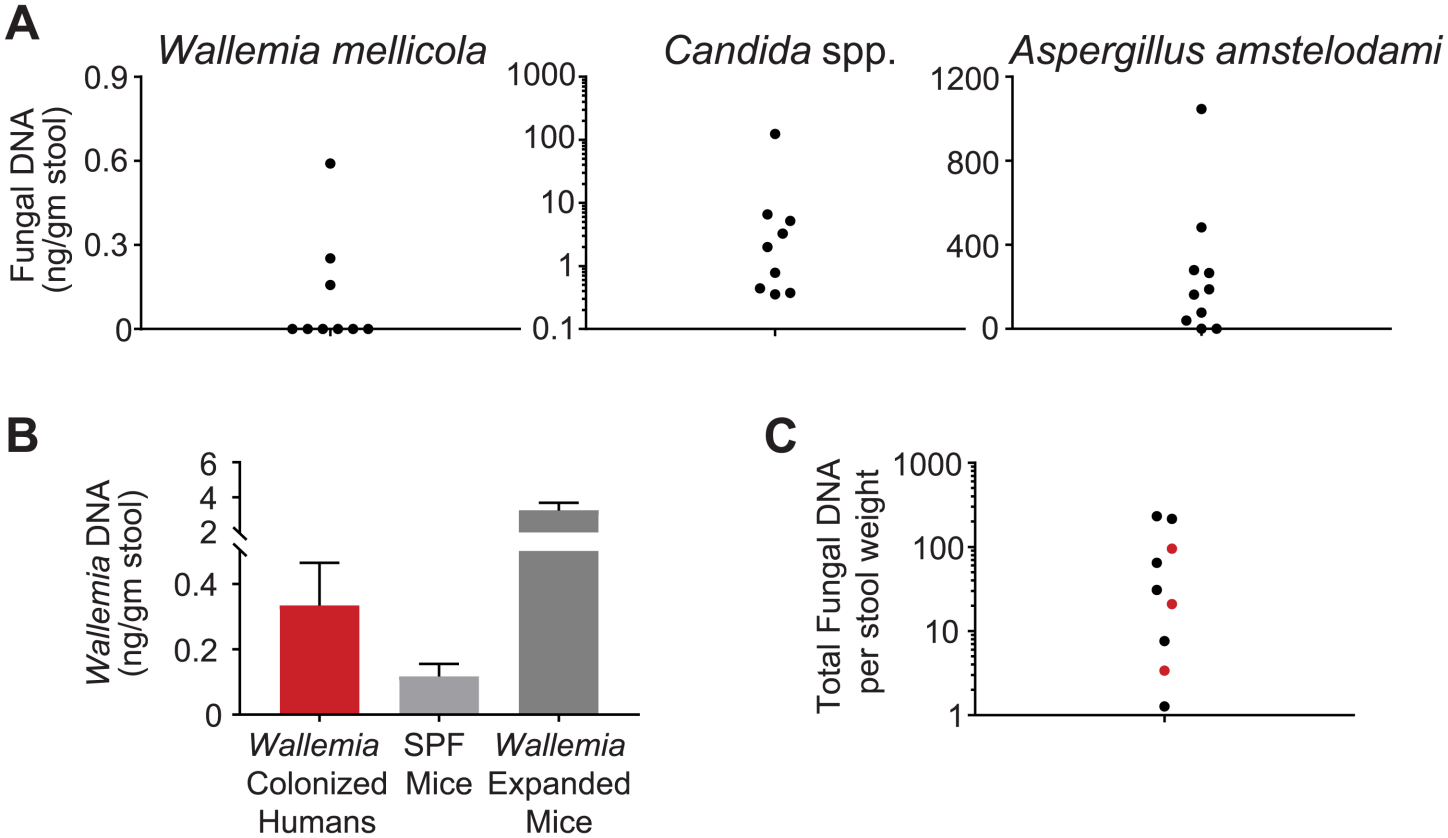
Commensal fungi examined in this manuscript are also detectable in the human gastrointestinal system. (**A**) Fungal DNA content per stool weight from healthy human volunteers with no recent antibiotic use. Levels of the indicated fungi were assessed by quantitative PCR of rDNA (by comparison to standard curves generated from control samples with defined fungal DNA content). (**B**) *Wallemia* abundance among the *Wallemia*-colonized humans is similar to unmodified SPF mice from our facility but less than mice with *Wallemia*-enhanced colonization. (**C**) Total fungal DNA burden in healthy human volunteers by rtPCR per stool weight. Red dots indicate human samples with detectable *Wallemia*. *Wallemia*-colonized humans do not generally have higher total fungal burden in this cohort compared to individuals without *Wallemia* colonization.

We next examined whether *W*. *mellicola* and the other commensal fungi studied in these experiments are found in the human gastrointestinal tract. Prior studies using ITS1 sequencing have detected sequences from *Wallemia* spp., *Aspergillus* spp., and *Epicoccum* spp. in human gastrointestinal samples, but species-specific PCR based assay of human gastrointestinal samples for the three fungal species discussed in this manuscript has not previously been described [28–30]. We extracted DNA from stool specimens from 9 healthy human subjects and performed rtPCR using species-specific primers to directly assess for DNA from each of the three relevant fungal species. We detected *Wallemia mellicola* in 3 of 9 human samples and *Aspergillus amstelodami* in 7 of 9 human samples (Fig 3A). We did not detect *Epicoccum nigrum* DNA in any human samples in our cohort, as no amplification was observed in any sample tested by rtPCR with *Epicoccum nigrum* specific primers. These results are consistent with prior studies showing that the composition of the human gut mycobiota varies across individuals and suggest that *Wallemia mellicola* and *Aspergillus amstelodami* may be capable of residing in the human gastrointestinal tract. We next examined the three human subjects who had *W*. *mellicola* DNA detectable in their stool. The total amount of *W*. *mellicola* DNA per stool weight was similar between *Wallemia*-colonized humans and SPF mice in our facility who are natively colonized with *W*. *mellicola* but less than mice who underwent the *Wallemia*-expanded colonization protocol (Fig 3B). This is not surprising because none of these healthy individuals carried a diagnosis of asthma or had recent antibiotic use, and we observed that antibiotic therapy was necessary to generate the *Wallemia* expansion dysbiosis in our mouse experiments. Human subjects found to be colonized with *Wallemia* do not have enhanced total fungal burden compared to non-*Wallemia*-colonized subjects (Fig 3C).

### Mice with expanded intestinal population of *Wallemia* demonstrate increased severity of house dust mite induced allergic airway disease

**Fig 4:**
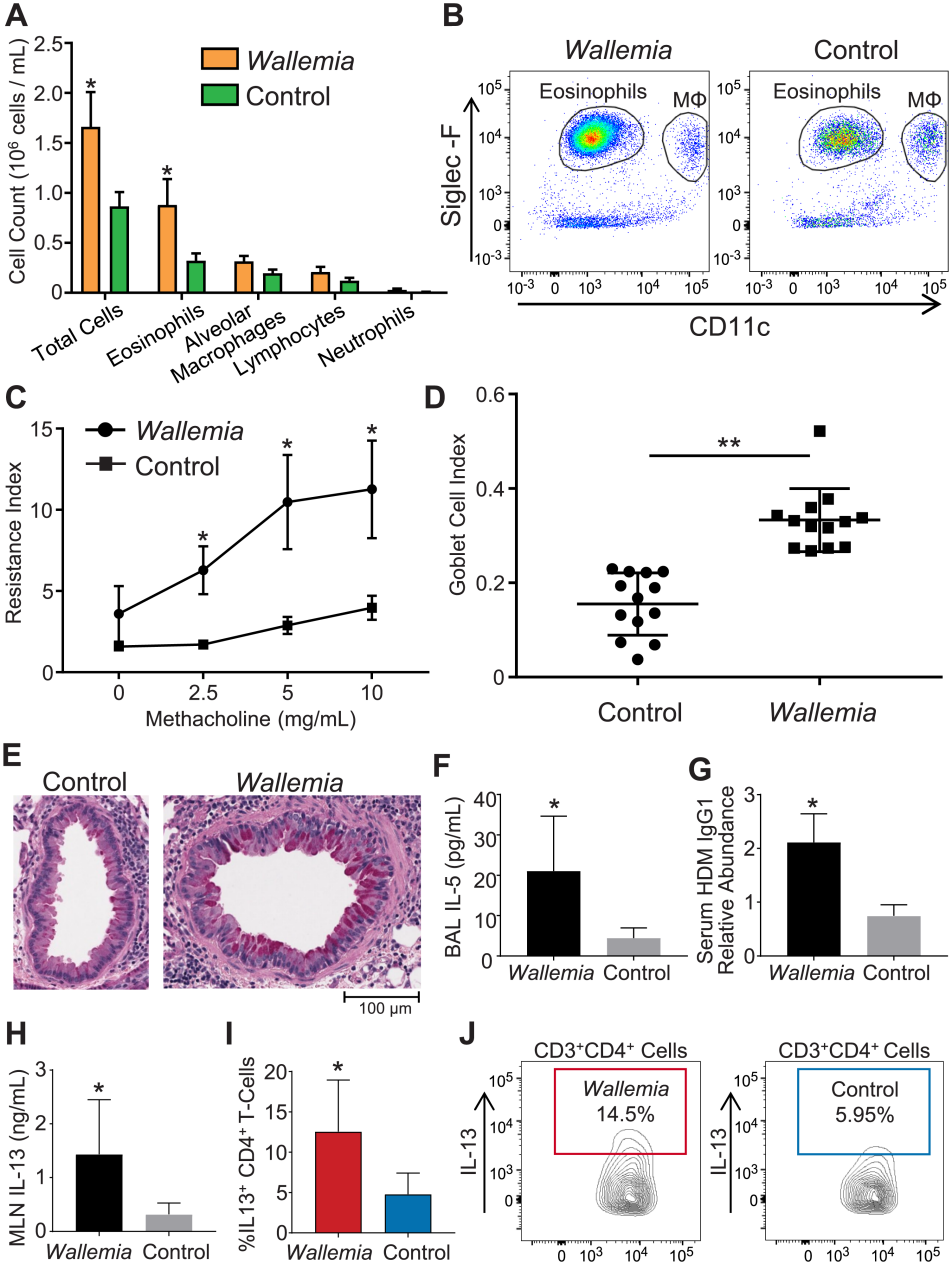
Expanded population of gastrointestinal *Wallemia* enhances the severity of house dust mite (HDM) induced allergic airways disease. (**A**) Airway eosinophilia in HDM-challenged mice is enhanced in *W*. *mellicola*-expanded animals. Control or *W*. *mellicola*-expanded mice were sensitized with intratracheal HDM once weekly for three weeks (n = 5 mice per group). Bronchoalveolar lavage (BAL) was performed and the presence of the indicated cells types were measured by flow cytometry. (**B**) Representative flow cytometry from BAL demonstrating enhanced eosinophilia. MΦ = macrophages. (**C**) *Wallemia*-expanded mice have enhanced airways hyperresponsiveness (AHR) in response to nebulized methacholine challenge compared to control mice after HDM sensitization. Airway resistance index measured in cmH_2_0xsec/mL. (**D**) *Wallemia*-expanded mice had enhanced goblet cell hyperplasia after HDM sensitization compared to control mice. Tissue sections were Periodic acid-Schiff (PAS) stained (400 x). (**D-E**) Goblet cell numbers were quantified. Figure illustrates combined result of three experiments. (**F**) IL-5 cytokine levels were measured in BAL samples by ELISA. (**G**) HDM-specific IgG1 in serum was measured by ELISA. (**H-J**) Mediastinal lymphocytes extracted from *Wallemia*-expanded or control mice were cultured in vitro and restimulated with HDM. Supernatants and cells were collected 5 days after restimulation. Supernatant IL-13 was measured by ELISA. IL-13^+^ cells were identified by flow cytometry as CD3^+^CD4^+^ cells that are IL-13^+^ by intracellular staining after PMA/ionomycin stimulation. Experiments independently performed at least 3 times (n = 5–6 mice per group) with figures illustrating representative example unless otherwise indicated. *, p < .05; **, p < .01; ***, p<0.001; unpaired two-tailed Student’s T-test.

Having established a mouse model of sustained *W*. *mellicola* colonization of the gut, we next sought to determine whether mice with *Wallemia*-expanded intestinal dysbiosis have an altered immune response to inhaled aeroallergens. We generated mice with a *Wallemia*-expanded mycobiota as described above by gavage of *W*. *mellicola* conidia into antibiotic-treated animals. Control mice were housed and treated identically to the *Wallemia*-expanded mice including the antibiotic treatment, but they received sterile water gavage rather than *W*. *mellicola* conidia gavage. We induced allergic airways disease in both groups by weekly intratracheal house dust mite (HDM) sensitization. Mice with expanded intestinal population of *W*. *mellicola* had increased severity of allergic airways disease compared to control by multiple measures. *Wallemia*-expanded mice demonstrated markedly greater bronchoalveolar lavage (BAL) cellularity driven primarily by increased alveolar eosinophils (Fig 4A and 4B) along with enhanced airways hyperresponsiveness (AHR) to methacholine challenge (Fig 4C). Histological analysis of the lungs showed enhanced goblet cell hyperplasia in *Wallemia*-expanded mice compared to controls (Fig 4D and 4E), and these mice also had higher BAL levels of IL-5 and serum IgG1 to HDM detectable at the end of the experiment compared to controls by ELISA (Fig 4F and 4G). To determine whether this effect on allergic airways disease might possibly be due to the initial bolus of *Wallemia* conidia, rather than the sustained colonization, we compared live *W*. *mellicola* gavage to gavage with heat-killed organisms. Heat-killed organisms did not influence allergic airways disease (S2A Fig). Similarly, we observed that an initial gavage with live *W*. *mellicola* did not influence disease if no pretreatment with antibiotics was provided to allow for sustained colonization (S2B Fig). Finally, to be certain that established colonization was essential, we delayed beginning the HDM sensitization for a week after the live *W*. *mellicola* gavage and found that the presence of *Wallemia* still exacerbated disease (S2C Fig). Together the data support the conclusion that enhanced colonization with live *W*. *mellicola* in the intestines exacerbates susceptibility to HDM allergic airways disease.

To begin to understand the mechanism by which expansion of gastrointestinal *W*. *mellicola* may have altered pulmonary immune response to HDM, we extracted the mediastinal lymph node from both groups and cultured lymphocytes in vitro. Five days after in vitro restimulation with HDM, lymphocytes extracted from mice with a *Wallemia*-expanded mycobiota had increased percentage of CD4^+^ T-cells positive for Th2 cytokine IL-13 by intracellular staining and increased supernatant IL-13 concentration (Fig 4H–4J). We did not observe any differences in IFNγ or IL-17 between the two groups by either intracellular staining or supernatant cytokine levels, suggesting that the *Wallemia*-expanded mycobiota has little effect on Th1 and Th17 response in this setting (S3 Fig). Finally, to determine whether this augmented pulmonary immune response could be due to stimulation by direct migration of *W*. *mellicola* to the lungs, we examined for the presence of *W*. *mellicola* in the lungs by multiple methods. We plated homogenized lung from *Wallemia*-expanded mice on antibacterial treated SDB agar plates and observed no growth of *W*. *mellicola*. We also performed PCR of whole lung homogenate and no amplification was observed in any sample tested by rtPCR using *W*. *mellicola* specific primers.

Together, these results suggest that an intestinal dysbiosis state characterized by enhanced presence of *W*. *mellicola* has a distant effect on pulmonary immune response characterized by increased eosinophilic airway inflammation, goblet cell hyperplasia, and enhanced secretion of IL-13 by mediastinal lymphocytes in response to HDM.

### Expansion of *Wallemia mellicola* alters intestinal bacterial and fungal communities

We have shown population expansion of intestinal *W*. *mellicola* enhances the severity of allergic airways disease in response to HDM allergen challenge. However, these experiments are insufficient to establish that intestinal *Wallemia* itself alters pulmonary immune response. Rather than having a direct effect, it is possible that *Wallemia* population expansion may alter or suppress commensal bacteria or other fungi which are themselves responsible for altering asthma severity. Notably, *Wallemia* species have been reported to secrete antibacterial compounds when grown in culture, so we hypothesized that expanded growth of *Wallemia* might alter bacterial community composition [31].

**Fig 5:**
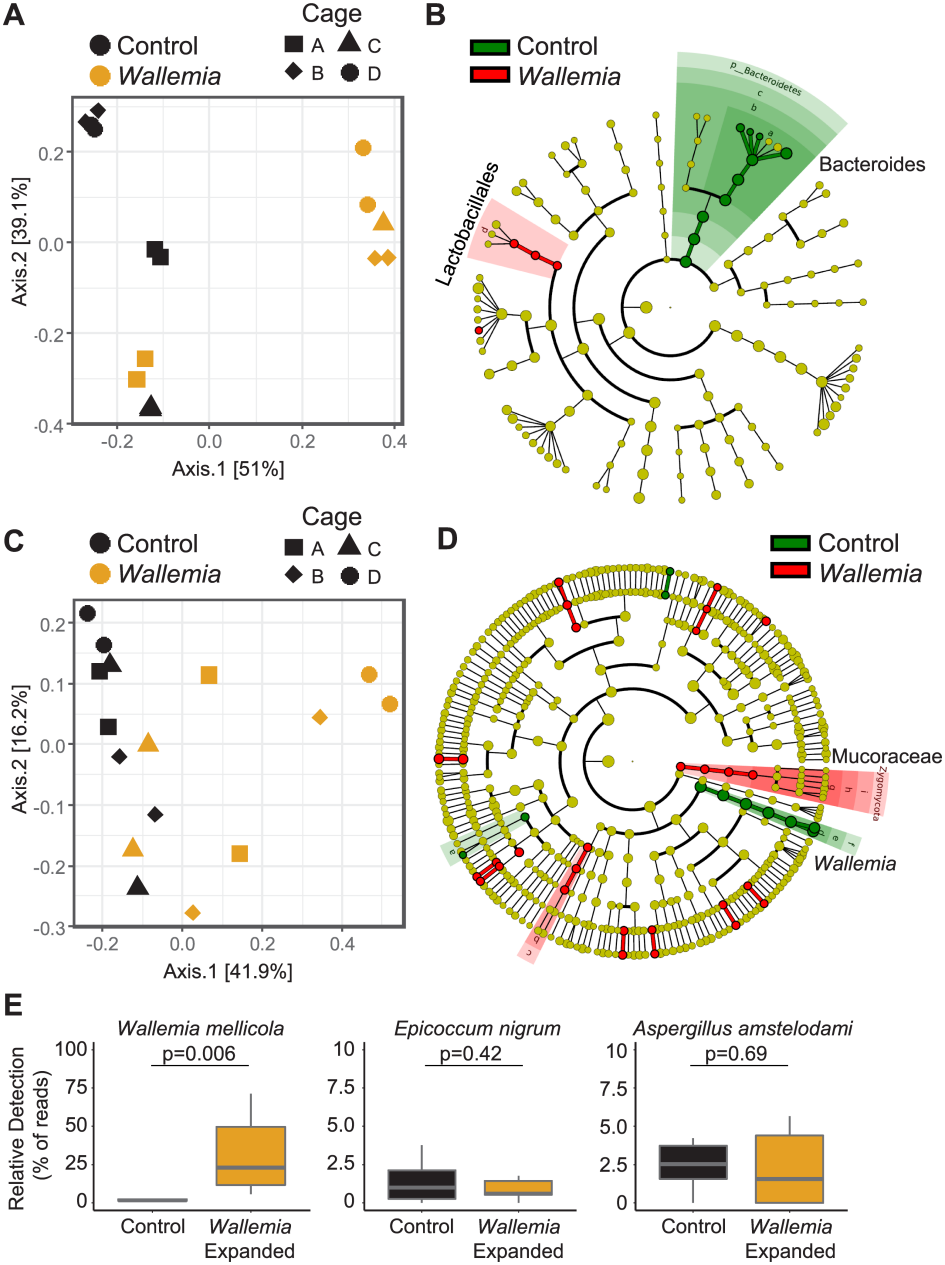
*Wallemia* expansion in specific pathogen free (SPF) mice alters commensal bacterial and fungal communities. (**A-B**) Bacterial communities are altered by *Wallemia* expansion. Plots show principle coordinate analysis and LEfSe analysis (cladogram) comparing bacterial communities by 16S sequencing in SPF mice 6 days after control or *W*. *mellicola* gavage as in Fig 1. Significant differences are highlighted on the cladogram (**C-D**) Fungal communities are altered by *Wallemia* expansion. Plots show principle coordinate analysis and LEfSe analysis (cladogram) comparing fungal communities by ITS1 sequencing in SPF mice 6 days after control or *W*. *mellicola* gavage as in Fig 1. (**E**) Box plots show relative abundance’s median and the first and third quartiles for the indicated fungi. Whiskers extend no further than 1.5 times IQR from the hinge, and p value determined as unpaired two-tailed Student’s T-test.

To determine whether expanded *W*. *mellicola* colonization altered bacterial and fungal communities, we analyzed fecal bacterial and fungal communities in *Wallemia*-expanded mice via 16S and ITS1 rDNA sequencing respectively. To control for potential cage-effects [32], each group (n = 8) was spread across 4 independent cages. Principal coordinates analysis (PCoA) and pair-wise differential abundance analysis with LEfSe [33] suggested that *W*. *mellicola* expansion altered bacterial communities (Fig 5A, 5B, S4A and S4B Fig). Further, we observed changes to fungal communities in *Wallemia*-expanded mice in addition to the expected expanded *W*. *mellicola* population (Fig 5C, 5D, S4C and S4D Fig). However, enhanced colonization with *W*. *mellicola* did not affect levels of *E*. *nigrum* or *A*. *amstelodami* (Fig 5E). Together, the data suggest that enhanced growth of *Wallemia mellicola* in the gut alters intestinal bacterial and fungal populations, and this makes it difficult to conclude whether *Wallemia mellicola* itself is sufficient to alter allergic immune responses in the lung.

### Colonization of fungus-free Altered Schaedler Flora (ASF) mice with *Wallemia mellicola* enhances the severity of allergic airways disease without substantially altering the bacterial community

To investigate whether the presence of *W*. *mellicola* in the gastrointestinal microbiota itself is sufficient to alter airway response to allergens, we employed a gnotobiotic mouse model that offers more precise control of intestinal microflora. Altered Schaedler Flora (ASF) mice are gnotobiotic animals with a stable microbiota consisting of eight defined bacterial species. Importantly for this study, they are fungus-free. Being colonized with bacteria, ASF mice are healthier than germ-free mice and have more mature immune systems [34–36].

**Fig 6:**
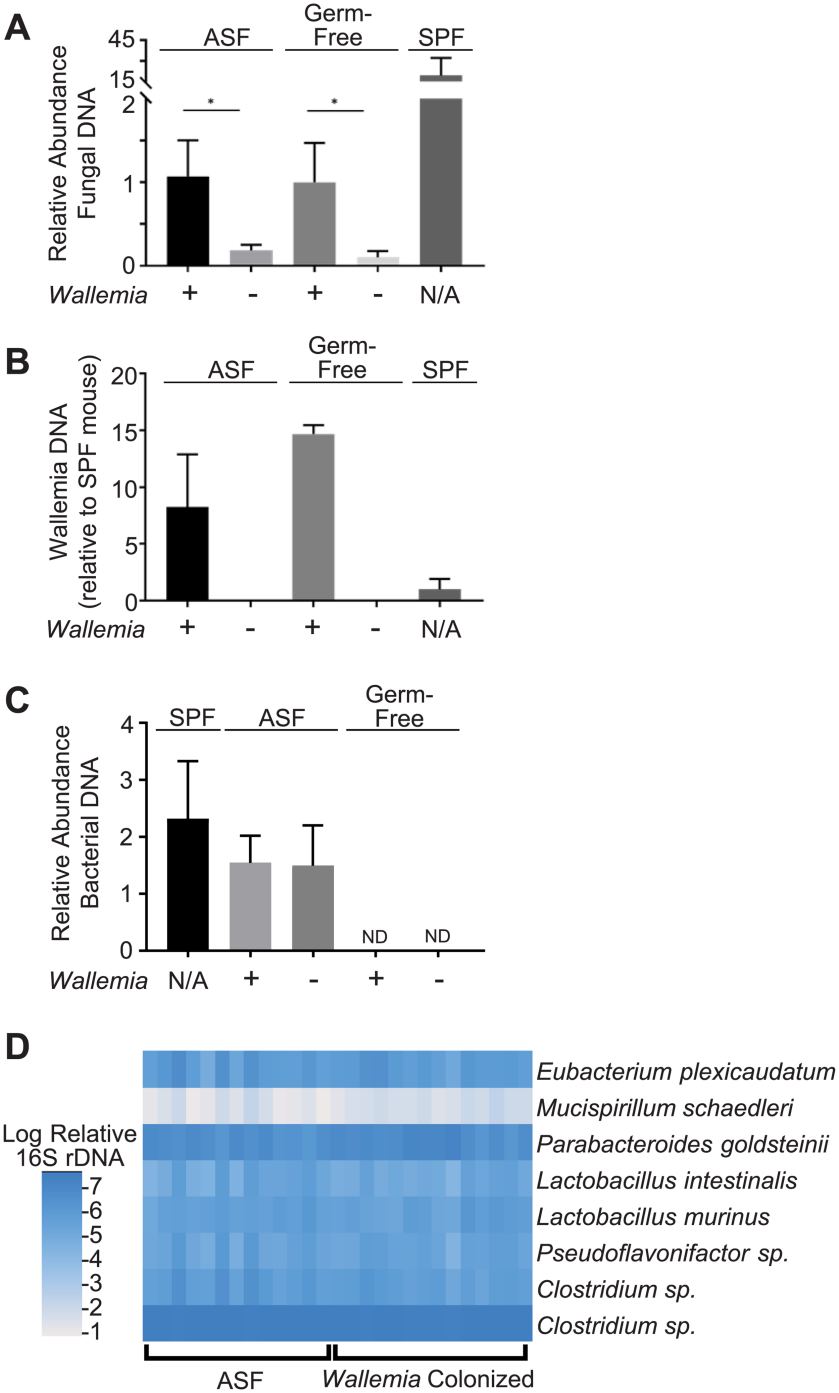
*W*. *mellicola* colonization of Altered Schaedler Flora (ASF) mice does not cause changes to ASF bacterial communities. (**A**) Total fungal burden was measured in fecal samples from the indicated animals (day 6 after fungal gavage as indicated) by quantitative PCR (n = 3–5 mice per group). Results reported as relative abundance of fungal DNA per stool weight. (**B**) Fecal *W*. *mellicola* levels were measured by quantitative rDNA PCR normalized to stool weight. SPF column depicts unmodified mice from our facility with native *W*. *mellicola* colonization. (**C**) Total bacterial abundance in fecal samples was assessed by quantitative PCR of 16S rDNA. Results reported as relative abundance of bacterial DNA per stool weight. (**D**) Heat map of relative abundance of the 8 ASF bacterial species in *Wallemia*-colonized ASF mice compared to control ASF mice. Figures are a representative example of experiment that was independently performed two times except panel D which depicts combined result of experiment that was independently performed three times. Statistical comparisons in panel A *, p < .05; **, p < .01; ***, p<0.001; unpaired two-tailed Student’s T-test. MANOVA analysis of heat map data depicted in panel D demonstrate no significant differences between groups (p>0.05).

While ASF mice and germ-free mice are initially “fungal-free”, gastric gavage with live *W*. *mellicola* is sufficient to establish fungal colonization (Fig 6A and 6B). *W*. *mellicola* grows similarly in both types of animals, although the total fungal burden remains substantially lower than SPF mice from our facility. Interestingly, *W*. *mellicola* colonization of ASF animals did not require prior antibiotic-mediated depletion of bacteria suggesting that the ASF bacteria do not substantially compete for the niche required by this fungus. We further observed that colonization of ASF animals with *W*. *mellicola* did not alter ASF bacterial microbiota. ASF mice have fecal bacterial levels similar to SPF mice, and colonization of ASF mice with *W*. *mellicola* did not grossly alter the total bacterial burden (Fig 6C). Upon measuring fecal levels of each of the 8 constituent ASF bacteria, we did not observe any *Wallemia*-induced quantitative changes in the population (Fig 6D, S5A and S5B Fig), although these results do not exclude the possibility that *W*. *mellicola* may alter metabolic products produced by the ASF bacterial community. Together the data suggest that these animals are a strong model for evaluating the specific ability of intestinal *W*. *mellicola* to influence allergic airways disease.

**Fig 7:**
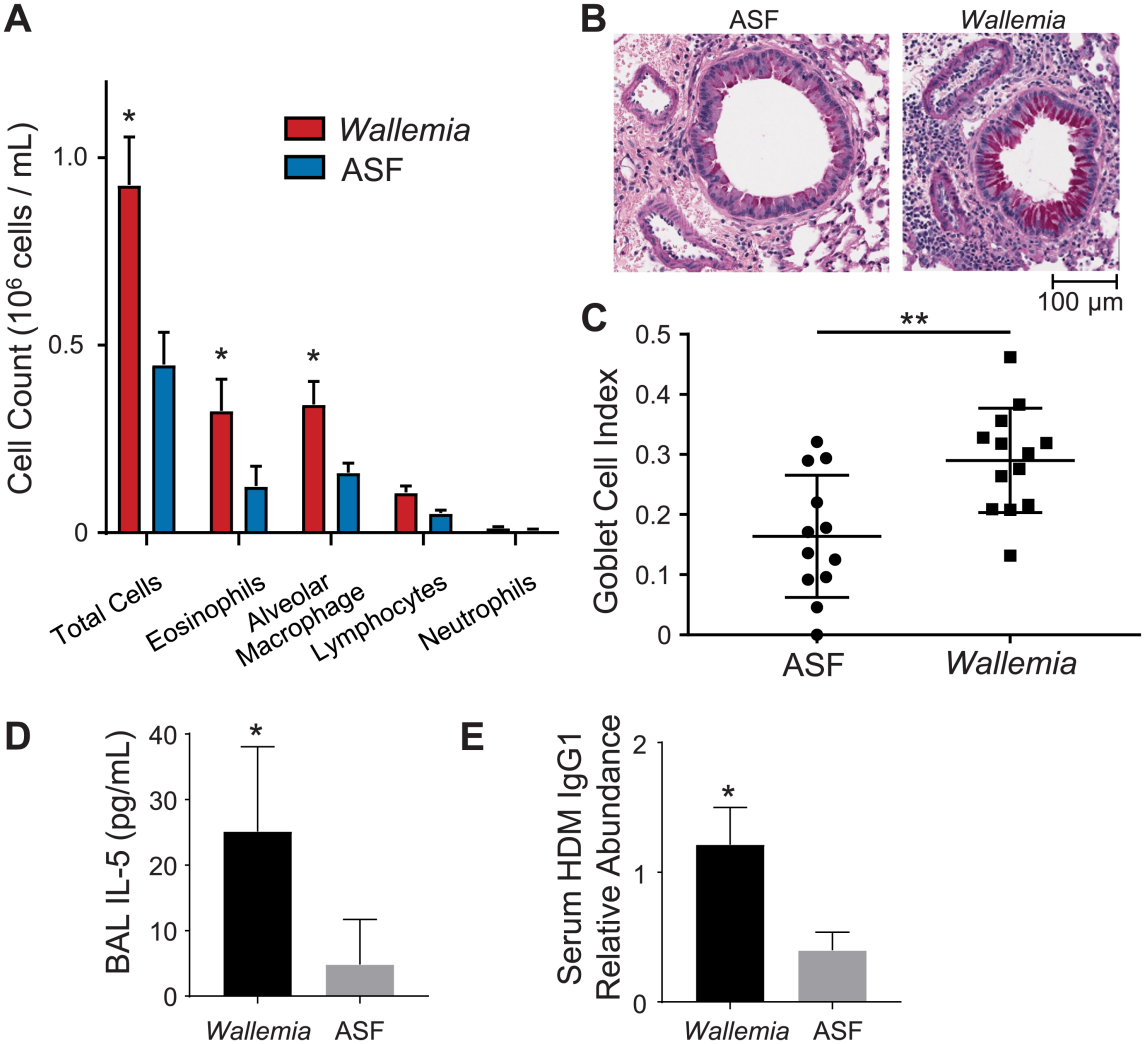
*Wallemia*-colonized Altered Schaedler Flora (ASF) mice have enhanced severity of allergic airways disease after HDM stimulation. (**A**) Control or *W*. *mellicola*-colonized ASF mice (n = 4–5 per group) were sensitized with HDM and challenged once weekly for three weeks. Bronchoalveolar lavage (BAL) was performed and the presence of the indicated cells types was measured by flow cytometry. (**B**) *Wallemia*-colonized ASF mice had enhanced goblet cell hyperplasia after HDM sensitization compared to control mice. Tissue sections were Periodic acid-Schiff (PAS) stained (400 x). (**C**) Goblet cell numbers were quantified. (**D** IL-5 cytokine levels were measured in BAL samples by ELISA. (**E**) HDM-specific IgG1 in serum was measured by ELISA. Figures are a representative example of experiments that were independently performed three times. *, p < .05; **, p < .01; ***, p<0.001; unpaired two-tailed Student’s T-test.

We next performed intratracheal HDM sensitization on these mice to induce allergic airways disease. We compared ASF mice to ASF mice colonized with *W*. *mellicola*. Like conventional mice with an expanded population of *W*. *mellicola*, we observed that colonizing ASF with *W*. *mellicola* increased the severity of HDM-induced allergic airways disease. Interestingly ASF mice demonstrated a mildly blunted response to HDM at baseline compared to SPF mice, but the pattern of disease was like that observed in SPF mice with antibiotic-associated expansion of *Wallemia*, including increased eosinophilic airway infiltration, increased histological inflammation with hyperplasia of mucous producing goblet cells, elevated BAL IL-5, and increased serum IgG1 to HDM (Fig 7). The data suggest that intestinal *W*. *mellicola* is sufficient to exacerbate allergic airways disease since the effect occurs without substantial alteration to other commensal bacteria.

## Discussion

We have shown that altered composition of the gastrointestinal mycobiota enhances the severity of allergic airways disease with enhanced eosinophilic airway inflammation and increased IL-13 production by mediastinal lymphocytes in response to HDM allergen stimulation. Interestingly, these effects are not due to a fungal overgrowth state where bloom of a single organism results in exponential population expansion of the total gastrointestinal fungal burden. Rather, the *W*. *mellicola* dysbiosis described herein is a shift in the composition of the commensal fungal community that occurs without substantial increase in the total fungal burden yet still produces a significant change in pulmonary immune response to inhaled allergens. The term “dysbiosis” is generally used to describe altered gut microbial ecosystem that results in negative host effects but is not an infectious state. We believe that the dysbiosis state described in this manuscript is not unique to *Wallemia mellicola*. Rather *Wallemia mellicola* dysbiosis may just be one representative example of a gut microbial pattern that alters pulmonary and systemic immune response. Other alterations of the gastrointestinal mycobiota community characterized by expansion of different fungal species may have distinctive beneficial or harmful effects on respiratory immune function.

The mechanism by which gastrointestinal *W*. *mellicola* population expansion alters pulmonary immune function is unknown, but there are several possibilities. *W*. *mellicola* may produce a toxin or metabolite that is absorbed, circulates systemically, then acts like a drug to modify the pulmonary immune response. This phenomenon has been observed with several other gut commensal microorganisms such as *Clostridium orbiscindens* which produces a small molecule metabolite (desaminotyrosine) that is systemically absorbed and alters pulmonary interferon response to influenza infection and prostaglandin E2 produced by intestinal *Candida* as discussed earlier [4, 5]. Interestingly, *Wallemia* species have been described to secrete a variety of mycotoxins such as walleminol, walleminone, and wallimidione when cultured in vitro [22–24]. Mycotoxins produced by other similar environmental fungal species have been shown to promote inflammatory cytokine secretion by lung alveolar macrophages [37]. Fungal mycotoxin production is generally influenced by the environmental conditions, and further study is needed to determine whether *Wallemia* produces toxins or other bioactive metabolites during growth in the mammalian intestine, whether these are absorbed into systemic circulation, and whether they affect host immune cells. It is also possible that *Wallemia* indirectly triggers production of different metabolites by the bacterial microbiota that then affects immune responses to challenge. Future studies will need to be designed to address this possibility.

Alternatively, *W*. *mellicola* may be recognized by the gastrointestinal host immune system and result in differential immune cell trafficking to the lungs. Gut commensal fungi have previously been shown to promote trafficking of different immune cell populations to non-GI organ systems. For example, migration of RALDH^+^ dendritic cells to peripheral lymph nodes in young mice is enhanced specifically by the presence of certain commensal gut fungal species [38]. *W*. *mellicola* is not generally considered to be a human pathogen, so there has been little prior study of immune response to this organism. *Wallemia* is known to elicit serum IgE and IgG responses [25, 39], but other innate and adaptive immune responses are not well characterized. Further study is needed to understand how *W*. *mellicola* is recognized by intestinal immune and epithelial cells and the consequences.

An important observation in this study is that oral antibiotic therapy places mice at risk for expansion of the dysbiosis-associated fungus *Wallemia mellicola*, but that mice with intact microbiota resist *W*. *mellicola* expansion after exposure. Our *W*. *mellicola* dysbiosis state in mice was established under conditions like those that a human asthma patient might experience. *Wallemia* are common environmental and food spoilage fungi, and therefore it is plausible that individuals may be exposed to live *Wallemia* conidia in their food or environment during a course of antibiotic therapy. Cefoperazone is a third-generation cephalosporin, and antibacterial medications in this class are widely used in patients with asthma and other respiratory diseases. Although antibacterials are not indicated for an uncomplicated asthma exacerbation, patients with severe asthma nonetheless receive frequent courses of broad spectrum antibacterial medications for a variety of indications throughout their lifetime [40, 41]. Furthermore, frequent courses of antibacterials, particularly early in life, have been associated with an increased incidence asthma [42, 43]. The concept that an intact microbiota resists colonization by pathogens such as *Clostridium difficile* has been previously established [44], and we now describe that a non-pathogenic commensal fungus can expand in the face of an antibiotic depleted microbiota and enhance the severity of allergic airways disease in mice. Intestinal fungal dysbiosis might therefore be an unrecognized but potentially important risk of each course of antibiotic therapy in patients with asthma and other respiratory disease. Further studies are needed to determine whether a phenomenon like the *Wallemia* dysbiosis state described in this manuscript can occur in humans during routine broad spectrum antibacterial therapy and more generally to what extent that gut mycobiota changes in humans alters pulmonary and systemic immune function.

## Materials and methods

### Ethics statement

All experiments involving research animals were performed in accordance with the recommendations outlined in the Guide for the Care and Use of Laboratory Animals. All research animal protocols were approved by the institutional animal use and care committee at Cedars-Sinai Medical Center (IACUC #6670 and #5160, PHS assurance number A3714-01). All studies involving humans and human samples were approved by the Cedars-Sinai Medical Center Institutional Review Board (IRB #0003358, Federalwide Assurance number 00000468). All human subjects were adults age >18 who provided written informed consent. All specimens were assigned an anonymized sample ID with no connection to patient identifiable information. Specimens were collected by the Cedars-Sinai MIRIAD IBD Biobank.

### Mice

7-8-week-old C57BL/6 female mice were purchased from Jackson Laboratory and housed in specific pathogen free conditions at the Cedars-Sinai animal facility. Germ-free mice C57BL/6 mice were obtained from Taconic Farms then housed and bred in microbially sterile flexible film isolators. A separate colony of altered Schaedler flora (ASF) mice was generated by gavage of live cultures of the 8 ASF bacterial species (Taconic Farms) into germ-free mice [35]. These mice were subsequently housed, bred, and raised in a separate flexible film isolator that was designated exclusively for maintenance of the ASF mouse colony. PCR based assays were performed to confirm stable presence of all 8 ASF bacterial species in subsequent generations of mice. ASF experiments were performed in age matched cohorts of 7-8-week-old male mice who had been born in a flexible film isolator to mothers colonized with ASF bacteria. Quality control testing including microbial culture and PCR was regularly performed on both the germ-free and ASF colonies to ensure that no contaminating microorganisms were introduced into either colony. Notably, ASF animals were verified to be fungus-free by FungiQuant rtPCR assays of both stool and environmental samples [45]. All animal experimental protocols were approved by the Institutional Animal Use and Care Committee (IACUC) at the Cedars-Sinai Medical Center (IACUC #5160 and #6670).

### Commensal fungal inoculation

Fungal cultures of *Wallemia mellicola* (ATCC 42694), *Aspergillus amstelodami (ATCC 46362)*, *Epicoccum nigrum* (ATCC 42773) were obtained from American Type Culture Collection (ATCC) and grown on Sabouraud dextrose agar for 7–14 days at 23°C as previously described [17]. Note that due to a recent taxonomic revision, the *Wallemia* strain examined in this study (ATCC 42694) was previously identified as *Wallemia sebi* but has been reclassified as *Wallemia mellicola* [19]. As documented in this manuscript, *Wallemia mellicola* is a naturally-occurring commensal microbe in mice and humans. Fungal conidia suspensions were generated by flooding a Sabouraud dextrose agar plate with mature fungal culture growth with 10 mL sterile water, gently washing by pipet to dislodge conidia, then passing the spore suspension through a 40 μm filter to exclude hyphal fragments. Conidia were centrifuged at 1600 rpm x4 minutes, resuspended in 1 mL sterile water, counted using hemocytometer, and the volume of water was adjusted to produce a 5 x 10^7^ conidia /mL suspension for gavage. For experiments involving heat-killed *Wallemia*, conidia were prepared as described and exposed to 95° C for 10 minutes then plated on Sabouraud dextrose agar to confirm non-viability.

### Antibiotic water treatment

Medicated drinking water was administered in selected experiments as follows: Fluconazole powder (Sigma-Aldrich, PHR1160) was dissolved in deionized water at a concentration of 0.5 mg/mL or cefoperazone sodium salt (Alfa Aesar, J65185) was dissolved in deionized water at a concentration of 0.5 mg/mL. The medicated water was provided as the exclusive source of drinking water for mice over the duration of antibiotic therapy, and mice were permitted to drink the medicated water ad libitum. The water was protected from light exposure by foil, and the medicated water was exchanged with freshly mixed solution every 3–5 days.

### Fungal expanded colonization

The fungal expanded colonization protocol described herein was adapted from experiments originally described by Noverr and Huffnagle [16]. SPF mice were treated with cefoperazone drinking water for 7 days to deplete intestinal bacteria. Mice subsequently received gavage of 5 x 10^6^ live conidia from a single species (*Wallemia mellicola*, *Aspergillus amstelodami*, or *Epicoccum nigrum*) or 5 x 10^6^ live yeast (*C*. *albicans*) suspended in 100 μL deionized water. Antibiotic drinking water was discontinued 4 hours after gavage. Stool collection was performed at several timepoints (Days #1, 7, 14, 21) and fungal DNA was extracted from stool as later described. Stool pellets were examined for consistency and for the presence of blood.

### House dust mite (HDM) sensitization experiments

Mice (n = 5 per group) first underwent the colonization protocol consisting of 7 days of cefoperazone 0.5 mg/mL followed by a single gastric gavage of either 5 x 10^6^ *Wallemia* spores suspended in 100 μL water (*Wallemia* colonization expansion group) or 100 μL sterile water (control group). Four hours after gavage, the cefoperazone water was discontinued in both groups and all mice were supplied with non-medicated drinking water for remainder of the experiment. Mice subsequently underwent house dust mite sensitization once every 7 days for a total of three treatments. *Dermatophagoides pteronyssinus* house dust mite suspension was purchased from Greer Labs and all mice in each experiment received HDM from the same lot. HDM sensitization was performed by first anesthetizing mice with isoflurane and then direct intratracheal administration of 100 μg HDM suspended in 50 μL of sterile saline. Mice were euthanized 48 hours after the final HDM treatment. Bronchoalveolar lavage (BAL) was performed immediately after euthanizing by cannulating the trachea with a 20 gauge catheter, instilling 1 mL sterile saline into the trachea with visual confirmation of inflation of the bilateral lungs, and aspirating the return lavage fluid. This was repeated for a total of 4 x 1 mL lavage for each mouse with >80% return (3.2 mL) defined as an adequate BAL. Cell suspension was counted using hemocytometer, then BAL solution was centrifuged at 1600 rpm x 4 minutes with supernatant removed for ELISA analysis and cell pellet resuspended in 1 mL FACS buffer for flow cytometry staining. Blood was aspirated by direct cardiac puncture, allowed to clot, then centrifuged at 6000 rpm x6 minutes to extract serum. A single lung lobe was fixed in formalin for H&E and PAS staining. To assess for *Wallemia* presence in the lungs, in separate experiments otherwise performed identically but with no BAL and n = 3 mice per group, the entire bilateral lung was resected en-bloc, the lung was suspended in 500 μL sterile PBS, morsellized with sharp scissors using sterile technique, and split to be plated in Sabouraud Dextrose Agar (SBD) with ampicillin 50 μg/mL for culture or homogenized with 0.5 mm bead and then processed for DNA extraction as described below for *Wallemia* rtPCR analysis.

To perform the in-vitro restimulation experiments, the mediastinal lymph node was harvested after the house dust mite sensitization experiments described above. A single cell suspension was generated by digestion with 200 u/mg type 4 collagenase (Worthington) and DNase 1 (Sigma-Aldrich) for 30 minutes at 37°C followed by passage through a 70 μm filter. Cells were resuspended in RPMI-1640 medium (Cellgro) supplemented with 2% FBS, beta-mercaptoethanol, and penicillin/streptomycin at a concentration of 2.5 x 10^6^ cells/mL. Cells were distributed into 24 well tissue culture plates, stimulated with 25 μg HDM, then incubated x5 days at 37°C in a cell culture incubator with 5% carbon dioxide. After incubation, supernatants were collected for ELISA and cell surface staining for flow cytometry was performed as described below. Cells were then stimulated for 4 hours with 20 nM PMA (phorbol 12-myristate 13-acetate, Sigma-Aldrich) and 1.3 μM ionomycin (Sigma-Aldrich) in the presence of GolgiStop (BD Biosciences), permeabilized, and then intracellular staining was performed as subsequently described.

Airway hyperresponsiveness to methacholine was measured 48 hours after the final HDM challenge. To perform this measurement, *Wallemia*-expanded and antibiotic only control mice were assessed using a Buxco FinePointe resistance and compliance system (Harvard Bioscience). Sodium pentobarbital (40 mg/kg i.p.; Butler Co.) was used to anesthetize mice before tracheostomy tube placement for mechanical ventilation. Within the sealed plethysmograph mouse chamber, transpulmonary pressure (i.e., Δ tracheal pressure -Δ mouse chamber pressure) and inspiratory volume were continuously monitored online by an adjacent computer. Airway resistance index was measured and calculated via the manufacturer supplied software (Buxco). After a baseline period in the Buxco apparatus, anesthetized and intubated mice received escalating doses (0 mg/mL, 2.5 mg/mL, 5 mg/mL, 10 mg/mL) of methacholine suspended in PBS by nebulizer (20 μL volume) over 30 seconds, and airway responsiveness to this bronchoconstrictor was measured over 3 minutes with an additional 1 minute for return to baseline before administration of next methacholine dose. At the end of the assessment of airway responsiveness each mouse was euthanized by exsanguination.

### Flow cytometry and intracellular cytokine staining

Fluorophore-conjugated antibodies directed against each of the following molecules were used to perform cell surface staining of BAL samples: Siglec-F (E50-2440, PE, BD Bioscience), CD11c (N418, violetFluor 450, Tonbo Bioscience), CD11b (M1/70, Alexa Fluor 700, eBioscience), Ly-6G (RB6-8C5, PE-Cy7, Tonbo Bioscience), CD3e (145-2C11, APC, eBioscience) CD19 (APC, eBioscience). BAL CD3^-^CD19^-^Siglec-F^+^CD11c^-^ cells were classified as eosinophils and CD3^-^CD19^-^Siglec-F^+^CD11c^+^ cells were classified as alveolar macrophages [46]. Antibodies directed against the following were used to stain mediastinal lymphocytes CD3e (145-2C11, PerCP-Cy5.5, eBioscience), and CD4 (GK1.5, FITC, BioLegend), and intracellular staining was performed after cell membrane permeabilization with saponins (PermWash Buffer, BioLegend) per manufacturer instructions using fluorophore-conjugated antibodies directed against IL-13 (eBio13A, PE-Cy7, eBioscience), IFNγ (XMG1.2, APC, eBioscience), and IL-17 (eBio17B7, PE, eBioscience). Sample data was acquired with a LSRII (BD Biosciences) and data was analyzed with FlowJo version 10.4.2 (BD Biosciences).

### ELISA

Supernatant and BAL cytokine measurements were performed using commercial IL-13 (eBioscience), IL-17 (BioLegend), IFNγ (BioLegend), and IL-5 (BioLegend) ELISA kits. All commercial ELISA assays were performed according to the manufacturer’s supplied instructions. To measure serum HDM-specific IgG1, a 96-well plate (Corning Costar 3361) was first coated overnight at 4°C with 25 mg/ml HDM in PBS. After plate washing, serum samples and controls were added and incubated at 23°C for 2 hours followed by washing and incubation with a detection biotinylated anti-mouse IgG1 (Biolegend, RMG1-1) at 1:750 dilution for 60 minutes. After washing, streptavidin-HRP (Biolegend) was added for 30 minutes. The plate was washed again, and the ELISA was developed with BD OptEIA TMB substrate set (BD Biosciences). OD measurements were recorded at 450–570 nm using a CLARIOstar (BMG Labtech) plate reader. A reference standard for HDM-specific IgG1 ELISAs was generated by pooling serum samples from HDM-sensitized mice, and varied dilutions of this standard were used to generate a standard curve. ELISA results were scaled such that this reference sample has a relative abundance of HDM-specific IgG1 of 1, and the same pooled reference standard was used for all HDM-specific IgG1 ELISAs reported in this manuscript.

### Histology

Whole mouse lung was perfused with phosphate buffered saline, resected en-bloc, and then fixed in 4% paraformaldehyde. Samples were embedded in paraffin, sectioned at 4 μm, and stained with hematoxylin and eosin or periodic acid-Schiff (PAS). Histological scoring was performed as previously described [47, 48]. Briefly, five airways were randomly selected on each PAS stained specimen slide: one primary airway, two separate secondary conducting airways, and two tertiary conducting airways. In each airway, one hundred sequential airway epithelial cells were identified and the number of PAS^+^ goblet cells in this segment were counted and divided by the total number of epithelial cells to generate the airway goblet cell index. The goblet cell index score for a specimen was the mean goblet cell index of the five examined airways. Prior to slide review and assignment of scores, all histology slides were assigned a random specimen ID such that the reviewer scoring each slide was blinded to the experimental group and to all specimen-associated experimental data. The blinding was broken only after the histological scoring was completed for all slides.

### Human commensal fungi studies

Human subjects were recruited after approval by Cedars-Sinai Medical Center Institutional Review Board (IRB #0003358). All volunteers self-reported as healthy with no chronic gastrointestinal or respiratory conditions and no acute illness at the time of study participation. A stool sample was collected from each volunteer and immediately frozen at −80°C. Samples were deidentified for this study by assigning a unique specimen ID number that was not connected to any subject demographic data. An approximately 50 mg portion of each frozen sample was processed for DNA extraction and rtPCR analysis using the same protocols as described for mouse stool specimens.

### Extraction of genomic DNA from organ tissue or fecal samples and rtPCR

DNA for rtPCR or fungal and bacterial sequencing was isolated from 1–2 fecal pellets following lyticase treatment, bead beating, and processing using QIAmp DNA mini kit (Qiagen) as previously described using a protocol optimized to lyse fungal cell walls for recovery of fungal DNA [11]. DNA extraction from organ tissue was performed by first homogenization tissue with a 0.5 mm steel bead in TissueLyser II (Qiagen) at frequency 30s^−1^ x4.5 minutes then proceeding per DNA extraction protocol used for stool samples. A primer-probe based detection system with iTaq Universal Supermix (BioRad) was used for FungiQuant and all species specific fungal rtPCR assays while the 16S, ASF, and pan-*Candida* rtPCR was performed using a SYBR Green supermix (BioRad) based assay. TaqMan rtPCR assays were run on an Eppendorf Mastercycler realplex2 machine using the following parameters: initial denaturation step of 95°C for 2 minutes followed by 45 cycles of 95°C x15s then 60°C x1 minutes. The SYBR green based rtPCR assay was run on an Eppendorf Mastercycler realplex2 machine using the following parameters: initial denaturation step of 95°C for 10 minutes followed by 40 cycles of 95°C x15s then 60°C x30s then 72°C x32s. The following primer pair and probe sequences were used for rtPCR assays: *Wallemia mellicola* F: GGC TTA GTG AAT CCT TCG GAG, *W*. *mellicola* R: GTT TAC CCA ACT TTG CAG TCC A, *W*. *mellicola* probe: 5’-(FAM) TGT GCC GTT (ZEN/) GCC GGC TCA AAT AG (3lABkFQ)-3’ [17]. *Aspergillus amstelodami* F: GTG GCG GCA CCA TGT CT, *A*. *amstelodami* R: CTG GTT AAA AAG ATT GGT TGC GA, *A*. *amstelodami* probe: 5’ (FAM) CAG CTG GAC (ZEN) CTA CGG GAG CGG G (3lABkFQ)-3’ [17]. *Epicoccum nigrum* F: TTG TAG ACT TCG GTC TGC TAC CTC TT, *E*. *nigrum* R: TGC AAC TGC AAA GGG TTT GAA T, *E*. *nigrum* probe: 5’-(FAM) CAT GTC TTT (ZEN) TGA GTA CCT TCG TTT CCT CGG C (3lABkFQ)-3’ [17]. FungiQuant F: GGR AAA CTC ACC AGG TCC AG FungiQuant R: GSW CTA TCC CCA KCA CGA FungiQuant probe: 5’-(FAM) TGG TGC ATG GCC GTT (3lABkFQ)-3’ [45]. 16S F: ACT CCT ACG GGA GGC AGC AGT, 16S R: ATT ACC GCG GCT GCT GGC. Pan-*Candida* F: GCA AGT CAT CAG CTT GCG TT Pan-*Candida* R: TGC GTT CTT CAT CGA TGC GA [49]. Detection of the ASF bacteria was performed using eight previously published organism-specific primer pairs [50]. A standard curve using dilutions of defined content of a pure sample of single species fungal DNA was created for PCR reactions to quantify their fungal DNA content.

### Microbiota profiling

Fungal ITS1 amplicons were generated in 20 μL PCR reactions using 3 μL of each sample with 35 cycles using Invitrogen Platinum SuperFi DNA Polymerase at an annealing temperature of 48°C using the primers ITS1F (CTTGGTCATTTAGAGGAAGTAA) and ITS2 (GCTGCGTTCTTCATCGATGC). Amplicons were then used in the second PCR reaction, using Illumina Nextera XT v2 barcoded primers to uniquely index each sample and 2x300 paired end sequencing was performed on the Illumina MiSeq, according to manufacturer’s instructions. Raw data processing and run de-multiplexing was performed using on-instrument analytics as per manufacture recommendations. Sequence reads from this study are available from the Sequence Read Archive under the project ID "PRJNA451226".

16S sequence data were processed and analyzed as previously described including OTU assignment by alignment with the GreenGenes reference database (release of May 2013) at 97% identity [51]. For analysis of ITS1 sequence data, raw FASTQ data were filtered to enrich for high quality reads including removing the adapter sequence by cutadapt v1.4.1 [52], truncating reads not having an average quality score of 20 over a 3 base pair sliding window, removing any reads that do not contain the proximal primer sequence or any reads containing a single N (unknown base) by a custom script. Filtered pair-end reads were then merged with overlap into single reads using SeqPrep v1.1 wrapped by QIIME v1.9.1 [53].

The processed high-quality reads were firstly aligned to previously observed host sequences (including rRNA, olfactory receptor and uncharacterized genes in human and mouse) to deplete potential contamination, then operational taxonomic unit (OTU) were picked by aligning filtered reads to the Targeted Host Fungi (THF) custom fungal ITS database (version 1.6) [11], using BLAST v2.2.22 in the QIIME v1.9.1 wrapper with an identity percentage ≥97%. 16S OTUs with average relative abundance >0.0001 and ITS1 OTUs with average relative abundance >0.001 were considered to be present for downstream analysis. PCoA of Bray–Curtis dissimilarities were evaluated and plotted using R package phyloseq v1.13.3 [54]. The pair-wise differential abundance analysis was conducted by using linear discriminant analysis with LEfSe v1.0.7 at default settings [33].

### Statistical analysis

Microbiota profiling analysis is described in detail above. Additional statistical analysis was performed using GraphPad Prism 7 (GraphPad Software) and JMP Pro 13.0.0 (SAS Institute Inc). Bar and line graphs report the mean value with error bars depicting the SEM unless otherwise specified.

## Supporting information

S1 Fig Gross and microscopic images of *Wallemia mellicola* grown in culture and growth of *Wallemia mellicola* from intestinal samples. (**A**) *Wallemia mellicola* colony growth on Sabouraud Dextrose agar. (**B**) Light microscopy images of cultured *Wallemia mellicola* spores and hyphae. *Wallemia mellicola* is a filamentous fungus which produces small 2–3μm spores. **(C)** *W*. *mellicola* colony forming units (CFU) cultured from stool pellet of germ-free mice mono-colonized with *W*. *mellicola*. Germ free mice received a single gavage of *Wallemia* live spores or sterile water control gavage (n = 5 mice per group) on day 1. After 10 days, stool pellets were collected, homogenized, and plated on Sabouraud dextrose agar, with a separate agar plate utilized for each individual mouse sample. Graph depicts *W*. *mellicola* colonies counted from each mouse specimen after 8 days of culture growth at 25°C. No other microbial growth was observed in either group. All experimental steps were performed using strict sterile technique, and mice received cage exchange (on day 3) into new sterile cages with fresh autoclaved food, water, bedding.
(TIF)

S2 Fig Comparison of HDM sensitization in alternate *Wallemia* inoculation models. (**A**) Live *Wallemia* is required for exacerbation of allergic airways disease. Each group received cefoperazone followed by gavage of live *W*. *mellicola* conidia, heat-killed *W*. *mellicola* conidia or sterile water gavage using the same protocol as depicted in Fig 1B and utilized for Fig 4 experiments. Heat-killed *W*. *mellicola* did not affect susceptibility to airways disease. (**B**) Antibiotic treatment is necessary for *Wallemia* exacerbated airways disease. Mice received either live *W*. *mellicola* conidia gavage or sterile water gavage with no cefoperazone therapy. As depicted in Fig 1D, cefoperazone treatment is necessary to facilitate *W*. *mellicola* intestinal population expansion, and mice with *W*. *mellicola* gavage alone did not demonstrate exacerbated allergic airways disease. (**C**) Delayed initiation of HDM sensitization after *Wallemia* gavage still results in exacerbated disease. Mice underwent the cefoperazone-facilitated *Wallemia* expansion protocol as illustrated in Fig 1B, but the initial HDM sensitization was performed 7 days after *Wallemia* gavage rather than immediately after gavage. For all experiments, each experimental group received once weekly HDM sensitization per protocol outlined in the methods with each group receiving the same dose and number of HDM treatments as utilized throughout this manuscript. Each graph depicts the bronchoalveolar lavage (BAL) cell count and differential 48 hours after final HDM dose. Each figure is a representative example of experiments that were independently performed twice with n = 4–5 mice per group. *, p < .05 by unpaired two-tailed Student’s T-test.
(TIF)

S3 Fig Mediastinal lymphocyte flow cytometry. (**A**) Flow cytometry gating used to identify CD3^+^CD4^+^ T-Cells in mediastinal lymph nodes. Results from intracellular staining of this population are presented in Fig 4H and 4I, S3B, and S3C. (**B**) IFNγ supernatant concentration and percentage T-cells positive for IFNγ after HDM in vitro restimulation of mediastinal lymphocytes. No statistically significant differences between groups. (**C**) IL-17 supernatant concentration and percentage T-cells positive for IL-17 after HDM in vitro restimulation of mediastinal lymphocytes. No statistically significant differences between groups. Supernatant and cells collected 5 days after restimulation. Supernatant cytokines measured by ELISA and cytokine intracellular staining performed on CD3^+^CD4^+^ cells after PMA/ionomycin stimulation. Figures are a representative example of experiments that were independently performed at least three times.
(TIF)

S4 Fig *Wallemia* expansion in specific pathogen free (SPF) mice alters commensal bacterial and fungal communities. Bacterial and fungal communities were evaluated by 16S and ITS1 rDNA amplicon sequencing respectively 7 days after control or *W*. *mellicola* gavage as in Fig 1. (**A-B**) Bacterial communities are altered by *Wallemia* expansion. Plots show (A) phylum-level distributions of bacteria in control and *Wallemia*-expanded animals as well as (B) linkage disequilibrium analysis scores (LDA, by LEfSe). (**C-D**) Fungal communities are altered by *Wallemia* expansion. Plots show (C) phylum-level distributions of fungi in control and *Wallemia*-expanded animals as well as (D) linkage disequilibrium analysis scores (LDA, by LEfSe). (A, C) Adjacent pairs of bars represent pairs of mice cohoused in the same cages.
(TIF)

S5 Fig *Wallemia* colonization in ASF mice does not alters bacterial communities. Relative fecal levels of each of the 8 bacterial constituents making up the Altered Schaedler Flora (ASF) were measured by quantitative PCR 7 days after control or *W*. *mellicola* gavage. (**A**) A stacked bar chart phylum-level distributions of bacteria in control and *Wallemia*-colonized ASF animals. (**B**) Principle coordinate analysis of Bray–Curtis dissimilarities is plotted. Dotted lines represent 95% confidence ellipses assuming a multivariate t-distribution.
(TIF)
