# Supplementary figures and images for: Expansion of commensal fungus Wallemia mellicola in the gastrointestinal mycobiota enhances the severity of allergic airway disease in mice
Source: PLoS Pathog. 2018 Sep 20;14(9):e1007260. doi: 10.1371/journal.ppat.1007260 (PMC6147580; doi:10.1371/journal.ppat.1007260)

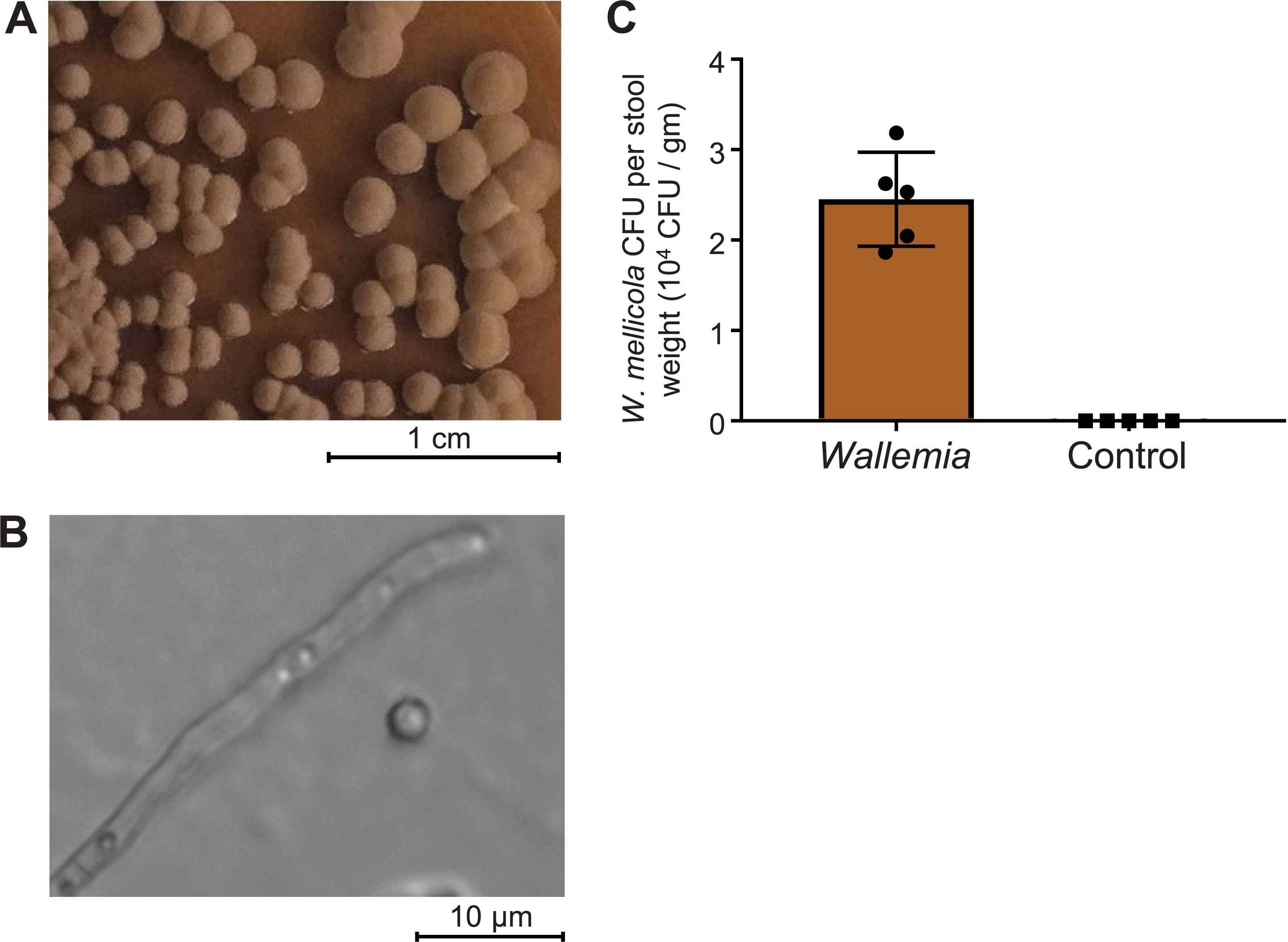

Supplement: S1 Fig — (A) Wallemia mellicola colony growth on Sabouraud Dextrose agar. (B) Light microscopy images of cultured Wallemia mellicola spores and hyphae. Wallemia mellicola is a filamentous fungus which produces small 2–3μm spores. (C) W. mellicola colony forming units (CFU) cultured from stool pellet of germ-free mice mono-colonized with W. mellicola. Germ free mice received a single gavage of Wallemia live spores or sterile water control gavage (n = 5 mice per group) on day 1. After 10 days, stool pellets were collected, homogenized, and plated on Sabouraud dextrose agar, with a separate agar plate utilized for each individual mouse sample. Graph depicts W. mellicola colonies counted from each mouse specimen after 8 days of culture growth at 25°C. No other microbial growth was observed in either group. All experimental steps were performed using strict sterile technique, and mice received cage exchange (on day 3) into new sterile cages with fresh autoclaved food, water, bedding. (TIF) [file ppat.1007260.s001.tif]

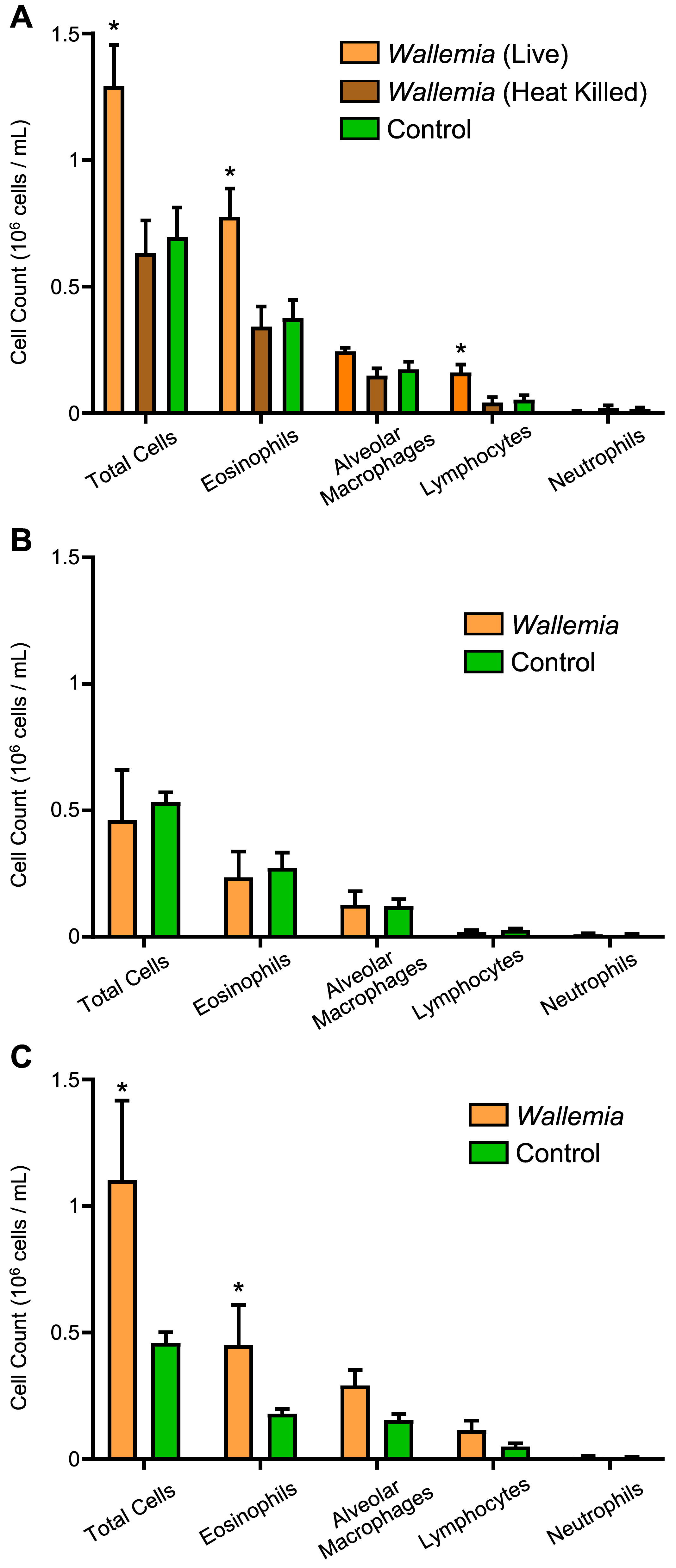

Supplement: S2 Fig — (A) Live Wallemia is required for exacerbation of allergic airways disease. Each group received cefoperazone followed by gavage of live W. mellicola conidia, heat-killed W. mellicola conidia or sterile water gavage using the same protocol as depicted in Fig 1B and utilized for Fig 4 experiments. Heat-killed W. mellicola did not affect susceptibility to airways disease. (B) Antibiotic treatment is necessary for Wallemia exacerbated airways disease. Mice received either live W. mellicola conidia gavage or sterile water gavage with no cefoperazone therapy. As depicted in Fig 1D, cefoperazone treatment is necessary to facilitate W. mellicola intestinal population expansion, and mice with W. mellicola gavage alone did not demonstrate exacerbated allergic airways disease. (C) Delayed initiation of HDM sensitization after Wallemia gavage still results in exacerbated disease. Mice underwent the cefoperazone-facilitated Wallemia expansion protocol as illustrated in Fig 1B, but the initial HDM sensitization was performed 7 days after Wallemia gavage rather than immediately after gavage. For all experiments, each experimental group received once weekly HDM sensitization per protocol outlined in the methods with each group receiving the same dose and number of HDM treatments as utilized throughout this manuscript. Each graph depicts the bronchoalveolar lavage (BAL) cell count and differential 48 hours after final HDM dose. Each figure is a representative example of experiments that were independently performed twice with n = 4–5 mice per group. *, p < .05 by unpaired two-tailed Student’s T-test. (TIF) [file ppat.1007260.s002.tif]

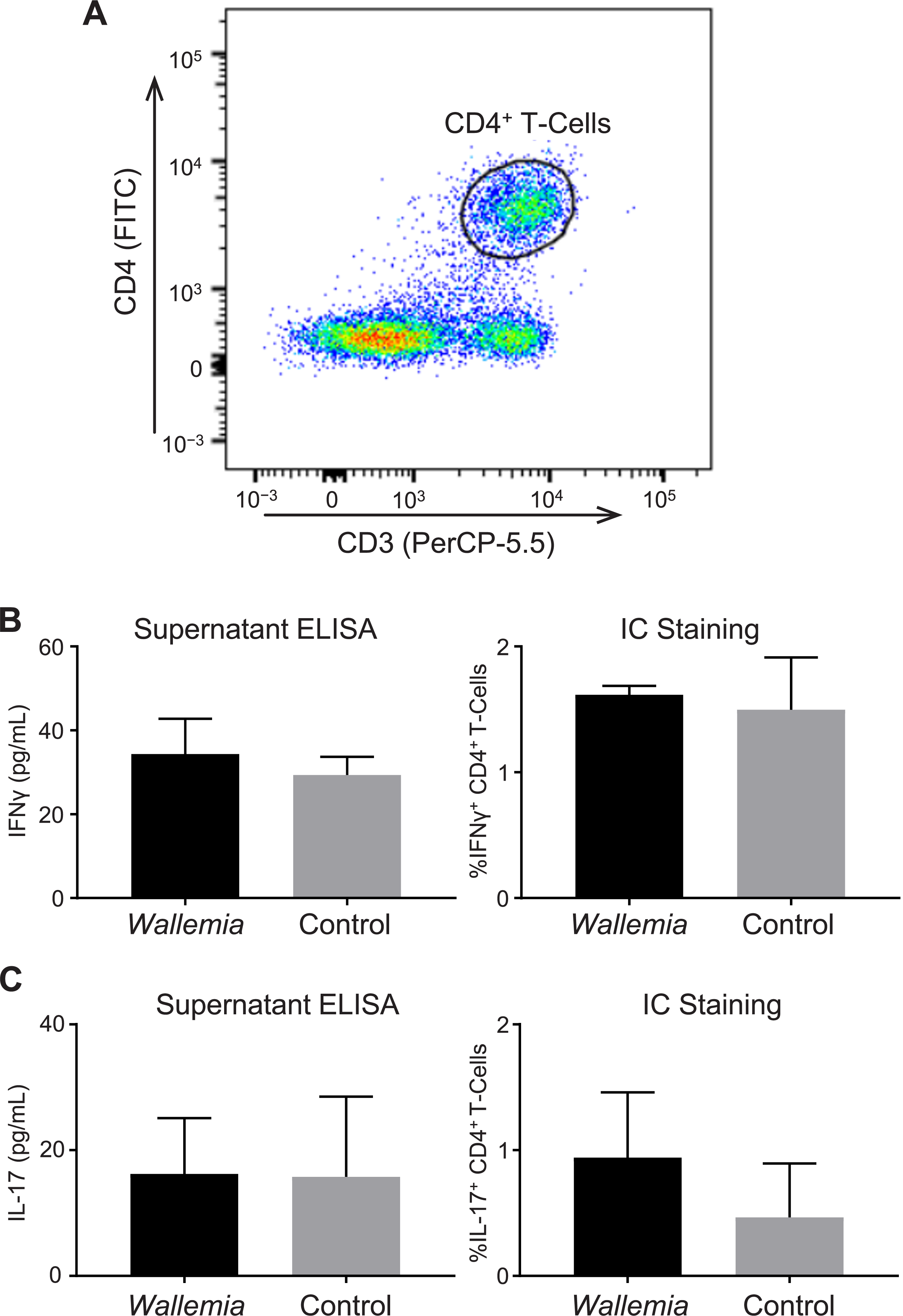

Supplement: S3 Fig — (A) Flow cytometry gating used to identify CD3+CD4+ T-Cells in mediastinal lymph nodes. Results from intracellular staining of this population are presented in Fig 4H and 4I, S3B, and S3C. (B) IFNγ supernatant concentration and percentage T-cells positive for IFNγ after HDM in vitro restimulation of mediastinal lymphocytes. No statistically significant differences between groups. (C) IL-17 supernatant concentration and percentage T-cells positive for IL-17 after HDM in vitro restimulation of mediastinal lymphocytes. No statistically significant differences between groups. Supernatant and cells collected 5 days after restimulation. Supernatant cytokines measured by ELISA and cytokine intracellular staining performed on CD3+CD4+ cells after PMA/ionomycin stimulation. Figures are a representative example of experiments that were independently performed at least three times. (TIF) [file ppat.1007260.s003.tif]

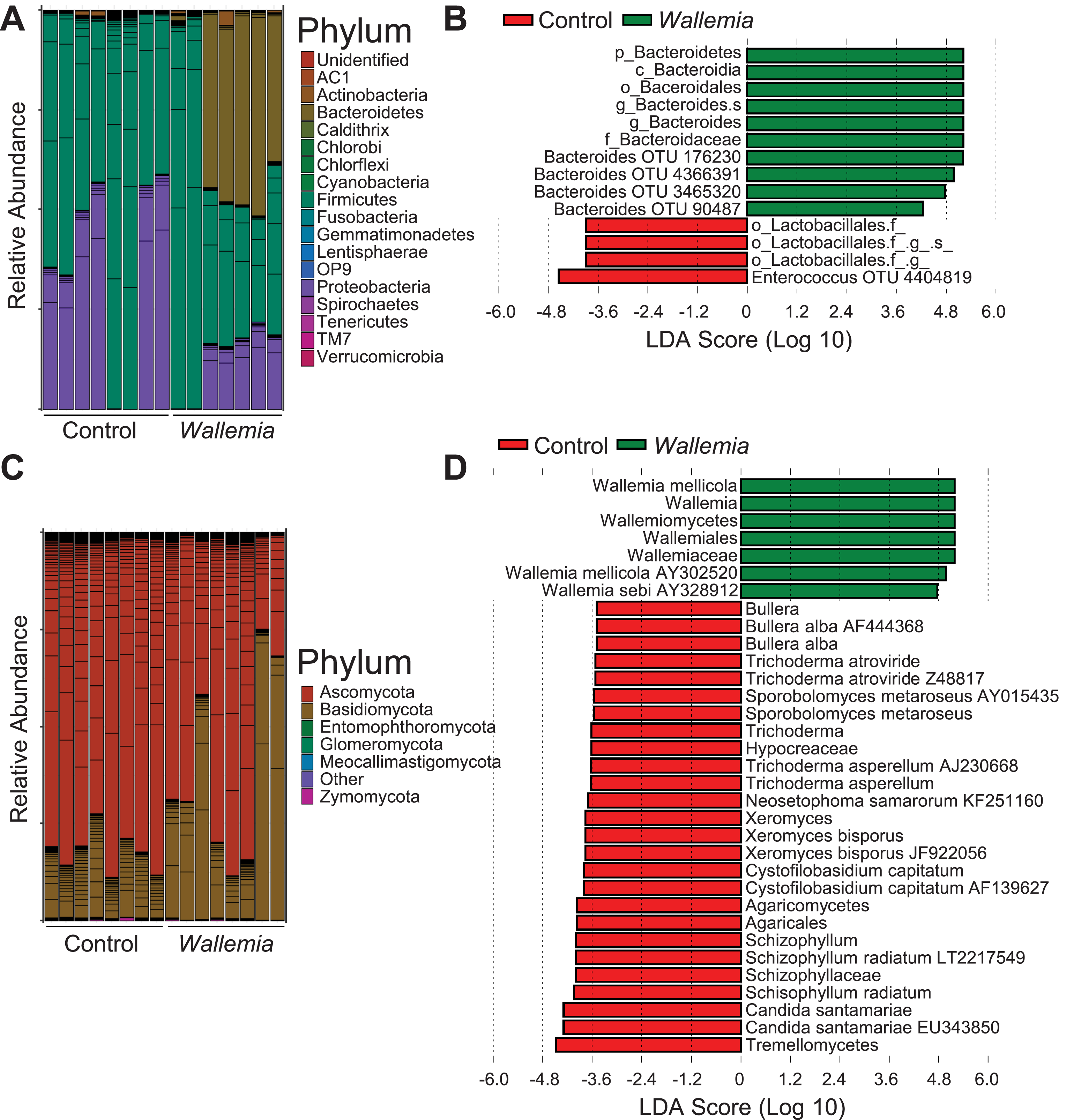

Supplement: S4 Fig — Bacterial and fungal communities were evaluated by 16S and ITS1 rDNA amplicon sequencing respectively 7 days after control or W. mellicola gavage as in Fig 1. (A-B) Bacterial communities are altered by Wallemia expansion. Plots show (A) phylum-level distributions of bacteria in control and Wallemia-expanded animals as well as (B) linkage disequilibrium analysis scores (LDA, by LEfSe). (C-D) Fungal communities are altered by Wallemia expansion. Plots show (C) phylum-level distributions of fungi in control and Wallemia-expanded animals as well as (D) linkage disequilibrium analysis scores (LDA, by LEfSe). (A, C) Adjacent pairs of bars represent pairs of mice cohoused in the same cages. (TIF) [file ppat.1007260.s004.tif]

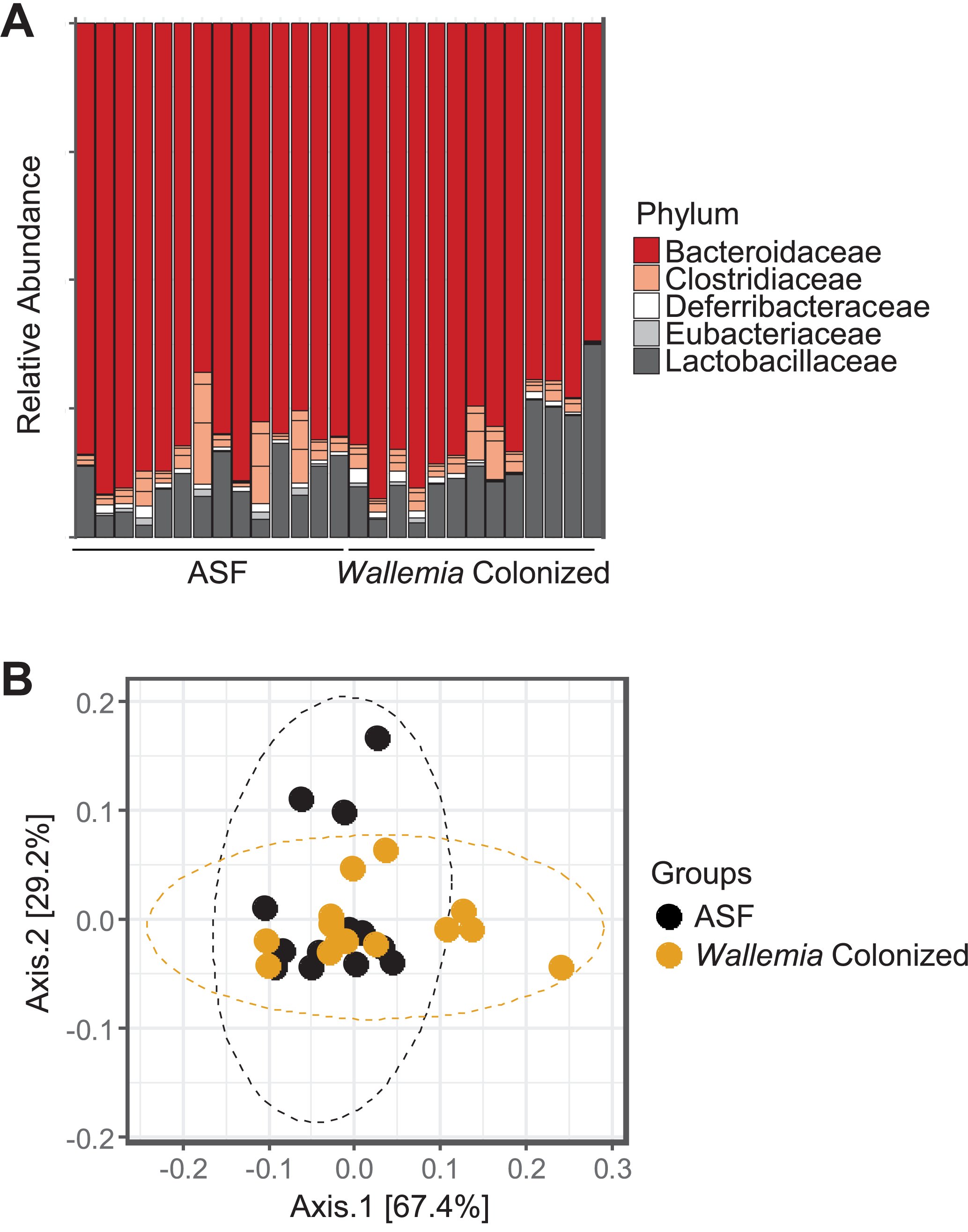

Supplement: S5 Fig — Relative fecal levels of each of the 8 bacterial constituents making up the Altered Schaedler Flora (ASF) were measured by quantitative PCR 7 days after control or W. mellicola gavage. (A) A stacked bar chart phylum-level distributions of bacteria in control and Wallemia-colonized ASF animals. (B) Principle coordinate analysis of Bray–Curtis dissimilarities is plotted. Dotted lines represent 95% confidence ellipses assuming a multivariate t-distribution. (TIF) [file ppat.1007260.s005.tif]
